# Pharmacological Inhibition of CBP/p300 Blocks Estrogen Receptor Alpha (ERα) Function through Suppressing Enhancer H3K27 Acetylation in Luminal Breast Cancer

**DOI:** 10.3390/cancers13112799

**Published:** 2021-06-04

**Authors:** Aaron Waddell, Iqbal Mahmud, Haocheng Ding, Zhiguang Huo, Daiqing Liao

**Affiliations:** 1 Department of Anatomy and Cell Biology, University Florida College of Medicine, UF Health Cancer Center, 2033 Mowry Road, Gainesville, FL 32610, USA; aawaddell@ufl.edu (A.W.); iqbalmahmud@ufl.edu (I.M.); 2 Departments of Biostatistics, University Florida College of Medicine, 2004 Mowry Road, Gainesville, FL 32610, USA; haochengding@ufl.edu (H.D.); zhuo@ufl.edu (Z.H.)

**Keywords:** breast cancer, estrogen receptor, CBP/p300 acetyltransferases, histone acetylation, bromodomain, enhancers, c-Myc, Cyclin D, A-485, GNE-049

## Abstract

**Simple Summary:**

Breast cancer is the most common cancer in women, affecting 1 in 20 women globally. Breast cancer is divided into four subtypes: luminal A, luminal B, HER2-enriched, and basal-like. Estrogen receptor-positive breast cancer accounts for nearly 80% of all breast cancer cases and most breast cancer deaths occur in patients with breast cancer of luminal subtypes. Estrogen receptor antagonists are the standard-of-care treatment for this type of breast cancer; however, resistance to these treatments is common in the clinic. Therefore, we aimed to explore novel therapeutic strategies for inhibiting estrogen receptor activity in breast cancer. Estrogen receptor relies on two highly related proteins, known as CBP and p300, to function. We report that CBP/p300 inhibitors effectively block estrogen receptor function and inhibit breast cancer cell growth. These findings reveal CBP and p300 as promising new targets for breast cancer treatment.

**Abstract:**

Estrogen receptor alpha (ER) is the oncogenic driver for ER+ breast cancer (BC). ER antagonists are the standard-of-care treatment for ER+ BC; however, primary and acquired resistance to these agents is common. CBP and p300 are critical ER co-activators and their acetyltransferase (KAT) domain and acetyl-lysine binding bromodomain (BD) represent tractable drug targets, but whether CBP/p300 inhibitors can effectively suppress ER signaling remains unclear. We report that the CBP/p300 KAT inhibitor A-485 and the BD inhibitor GNE-049 downregulate ER, attenuate estrogen-induced c-Myc and Cyclin D1 expression, and inhibit growth of ER+ BC cells through inducing senescence. Microarray and RNA-seq analysis demonstrates that A-485 or EP300 (encoding p300) knockdown globally inhibits expression of estrogen-regulated genes, confirming that ER inhibition is an on-target effect of A-485. Using ChIP-seq, we report that A-485 suppresses H3K27 acetylation in the enhancers of ER target genes (including MYC and CCND1) and this correlates with their decreased expression, providing a mechanism underlying how CBP/p300 inhibition downregulates ER gene network. Together, our results provide a preclinical proof-of-concept that CBP/p300 represent promising therapeutic targets in ER+ BC for inhibiting ER signaling.

## 1. Introduction

Breast cancer (BC) is the most common cancer in women and affects 1 in 20 women globally. In 2021, it is estimated that 281,550 women will be diagnosed with BC and 43,600 women will die of the disease in the United States. Significantly, ER+ BC accounts for 60–80% of BC cases and most BC deaths occur from ER+ BC. BC is a highly heterogeneous cancer and has been classified into four major molecular subtypes: luminal A, luminal B, HER2-enriched, and basal-like. These subtypes are distinguished through the expression of specific markers, such as ERα (or simply ER), progesterone receptor (PR), and human epidermal growth factor receptor 2 (HER2). Luminal A is ER+ and/or PR+, but HER2-; Luminal B is ER+ and/or PR+, and HER2+; HER2-enriched is ER- with genomic amplification of the ERBB2 gene encoding HER2; and basal-like is negative of ER, PR, and HER2, which is also known as triple-negative BC or TNBC.

ER is a steroid hormone nuclear receptor that functions as a transcription factor when activated by the potent estrogen 17β-estradiol (E2) [1,2]. Ligand-bound ER undergoes homodimerization and translocates to the nucleus where it binds estrogen response elements (EREs) in promoters and enhancers to regulate gene expression [1,2]. ER is required for the normal development of female mammary glands and the reproductive system, but is also a dominant driver of ER+ BC [3]. ER signaling promotes tumor progression through activation of oncogenes and regulation of other cellular functions that control cell survival and proliferation [4,5,6,7]. MYC (encoding the protooncogene c-Myc) and CCND1 (encoding Cyclin D1) are well-known ER target genes and are responsible for estrogen-induced cell cycle progression [4,5,6,7]. Therefore, ER+ BC is treated with endocrine therapies that inhibit ER signaling, which include aromatase inhibitors (e.g., exemestane), selective ER modulators (SERMs, e.g., tamoxifen) and selective ER degraders (SERDs, e.g., fulvestrant) [3,8,9,10,11]. However, de novo and acquired resistance to endocrine therapies are common in the clinic and alternative therapeutics are needed to improve clinical outcomes for patients with drug-resistant ER+ tumors [3,10,11,12,13,14]. Significantly, ER, c-Myc, and Cyclin D1 are known to play roles in driving tamoxifen resistance in BC cells in vitro and in patients. Indeed, tamoxifen-resistant BC cell lines have elevated expression of ER, c-Myc, and Cyclin D1 and inhibition of MYC restores tamoxifen sensitivity in these cells [15]. High MYC expression also correlates with poor relapse-free survival in patients treated with endocrine therapies [15]. Additionally, Cyclin D1 can activate ER function independently of estrogen, which apparently is not inhibited by anti-estrogen therapies [16]. Therefore, strategies that inhibit the downstream effectors of ER, such as Cyclin D1, have been extensively investigated. Cyclin D1 promotes G1 to S cell cycle progression through activating cyclin-dependent kinases (CDKs) 4 and 6. A number of CDK4/6 kinase inhibitors such as PD 0332991 [17] (Palbociclib), LY-2835219 (Abemaciclib), and LEE011 (Ribociclib) [18] in combination with an aromatase inhibitor have been approved by the FDA for treating patients with hormone receptor-positive and HER2-negative BC. Unfortunately, resistance to CDK4/6 inhibitors presents a critical clinical challenge [19]. 

Inhibition of ER co-activators is an alternative strategy that can block estrogen-dependent and -independent ER activity. ER relies on critical co-activators to upregulate the expression of its target genes, including MYC and CCND1 [1,20,21,22,23,24,25]. These co-activators include the steroid receptor coactivators (SRCs) (also known as nuclear receptor coactivators or NCOAs) and the CREB-binding protein (CBP)/E1A-associated protein p300 (p300) [21,22,23]. Targeting these ER co-activators shows promise as a new strategy for inhibiting ER activity and there have been increasing efforts to explore the therapeutic potential of inhibitors of these co-activators in BC [26,27]. Targeting hormone receptor co-activators has proven successful in other hormone-dependent cancer types [28,29,30,31,32,33,34,35,36]. CBP and p300 are two paralogous lysine acetyltransferases (KAT) that share a highly conserved modular structure and are often together referred to as CBP/p300 [37]. CBP/p300 are recruited to chromatin through an extensive interactome involving several hundred proteins [37], among which ER and SRCs are key CBP/p300 interactors [21,22,37]. CBP/p300 contains several folded domains along with intrinsically disordered regions that facilitate protein–protein interactions. The folded domains include the catalytic KAT and the acetyl-lysine binding bromodomain (BD) [37]. Through their KAT domain, CBP/p300 catalyze acetylation of histones, transcription factors, and many other proteins to regulate gene expression and other cellular functions [37,38]. All core histones are CBP/p300 substrates [38,39,40], and functional effects of the acetylation of some of the lysines, such as H3K27ac, in core histones have been extensively investigated. H3K27ac is specifically catalyzed by CBP/p300 and is important for active transcription [39,40,41,42,43]. The CBP/p300 BD binds acetylated lysine residues on transcription factors and histones, which facilitates the recruitment of CBP/p300 to the chromatin, thus allowing CBP/p300 to regulate gene expression and chromatin acetylation [44,45,46]. There have been continuous efforts to develop CBP/p300 catalytic and BD inhibitors to ablate CBP/p300 activity [27,29,30,37,47]. Potent and selective inhibitors of CBP/p300 KAT [29,47,48,49] and BD [30,50] have recently been discovered and characterized in preclinical studies [51]. Significantly, the CBP/p300 KAT inhibitor A-485 [29] and BD inhibitor CCS1477 [50] have antitumor activity in vivo in mouse models of prostate cancer and the latter also shows promising clinical activity in human patients with prostate cancer [50]. Importantly, A-485 is highly specific to CBP/p300 with a nanomolar potency and does not inhibit other KATs up to 10 µM [29]. 

Notably, pharmacological inhibition of CBP/p300 as a therapeutic strategy in ER+ BC remains largely unexplored, despite clear evidence that CBP/p300 serve as critical ER co-activators [20,21,22,23,24,52]. Structural studies suggest that an estrogen-activated ER homodimer recruits two SRC-3 molecules (one SRC-3 per ER monomer) and that the SRC-3 proteins then recruit p300 to the ER activation complex [21]. In this model, p300 indirectly interacts with the ER [21]. CBP is also an ER co-activator and probably interacts with the SRC proteins and the ER in a similar fashion, although a structure of the CBP/ER complex has not been determined [53]. It is now clear that the CBP/p300 histone acetyltransferase (HAT) activity is required for ER-mediated transcription and that p300 recruitment to the ER activation complex increases its HAT activity [21,22,23]. Additionally, in the ER activation complex, the p300 BD is free to interact with acetylated histones and CBP/p300 BD inhibitor CBP30 blocks recruitment of p300 to ER target genes [21]. These studies suggest both CBP/p300 KAT and BD represent promising targets for inhibiting ER function. 

In this study, we have investigated the effects of CBP/p300 KAT inhibitor A-485 and BD inhibitor GNE-049 on histone acetylation and the expression of ER target genes MYC and CCND1 in ER+ BC cell lines. We then globally analyzed the effects of A-485 and EP300 (encoding p300) knockdown on the expression of the estrogen-regulated gene network. We delineated the effects of A-485 on global H3K27ac deposition in MCF-7 cells and correlated H3K27ac levels with changes in gene expression. We assessed the effects of A-485 and GNE-049 on inhibiting growth of ER+ BC cell lines in vitro. Our data show both A-485 and GNE-049 potently and specifically suppress ER-mediated gene expression and inhibit proliferation of ER+ BC cells.

## 2. Materials and Methods

### 2.1. Analysis of CREBBP/EP300 Dependency and Expression in Cell Lines and Tumors 

EP300 and CREBBP gene effect data were downloaded from The Cancer Dependency Map (DepMap.org, accessed on 19 May 2021) for the CRISPR (DepMap 21Q2 Public, CERES) dataset [54]. Gene effect data for cell lines in the primary disease category were used in the analysis. The EP300 and CREBBP gene effect for the cell lines was then plotted in relation to the primary disease categories. EP300 and CREBBP gene expression data was downloaded from DepMap.org (accessed on 19 May 2021) for breast cancer cell lines in the Expression 21Q2 Public dataset [55]. CREBBP and EP300 gene expression values for each cell line were used to generate a scatterplot and the Pearson correlation coefficient (R) with its associated *p*-value is displayed for each plot. Data for the TCGA Breast Cancer (BRCA) dataset was obtained from the Xena database (xenabrowser.net, accessed on 18 February 2021) for primary tumors [56]. The mRNA expression values for CREBBP and EP300 were obtained from the BRCA dataset and transformed into Z-scores for generation of boxplots. ER status of the tumors was determined using the ER_Status_Nature2012 filter. A Welch’s *t*-test was performed for comparison between two groups. A one-way ANOVA followed by a post-hoc Welch’s *t*-test was performed when more than two groups were compared. mRNA expression values in (log2(norm_count+1)) units were also obtained for all primary tumor samples in the BRCA dataset and used to generate a scatterplot for a correlation between CREBBP and EP300 expression in BRCA tumors. The Pearson correlation coefficient (R) was calculated, and the associated *p*-value is displayed for each scatterplot.

### 2.2. TCGA CBP/p300 Interactome Analysis 

A list of CBP/p300 interaction partners was obtained from a publication [37] and modified to remove viral proteins and to include additional known partners (Table S1). This list of partners was then analyzed for their expression in breast cancer using data from The Cancer Genome Atlas Program (TCGA) [57]. The transcriptomic data in the TCGA dataset was downloaded from firehose (http://firebrowse.org, accessed on 22 May 2020). The gene expression levels were normalized using the RSEM method, followed by log transformation (i.e., log2(x + 1) where x is the RSEM value). We performed differential expression (DE) analysis using R package limma to compare normal tissues with breast cancer subtypes luminal A and B, respectively. Multiple testing was corrected by Benjamini–Hochburg method. The differentially expressed genes (q value < 0.05, |FC| > 1.25) were visualized via heatmaps. Genes that were found to be upregulated (q value < 0.05, FC > 1.25) were further analyzed by comparing the list of genes to Reactome Pathways using GSEA. Differentially expressed binding partners are shown in Table S1.

### 2.3. Reagents and Cell Culture

A-485 (HY-107455) and GNE-049 (HY-108435) were purchased from MedChemExpress (Monmouth Junction, NJ, USA). Cell lines used for this study were obtained from the ATCC (Manassas, VA, USA) and recently authenticated by LabCorp (Burlington, NC, USA). MCF-7, BT-474 and T-47D were grown at 37 °C with 5% CO_2_ in a humidified atmosphere. MCF-7 cells were cultured in Dulbecco’s Modified Eagle Medium (DMEM with 4.5 g/L glucose, L-glutamine and sodium pyruvate, Corning) supplemented with 10% bovine calf serum (HyClone, Cytiva, Marlborough, MA, USA), penicillin (10 units/mL), and streptomycin (10 µg/mL) (designated as complete media). T-47D and BT-474 cells were cultured in DMEM supplemented with 10% fetal bovine serum (R&D Systems, Inc., Minneapolis, MN, USA), penicillin (10 units/mL), and streptomycin (10 µg/mL) (designated as complete media). Estrogen deprivation was achieved through washing cells with phosphate-buffered saline (PBS, without calcium and magnesium, Corning) and culturing cells for 24 h in phenol red free DMEM (21063-029, Gibco, ThermoFisher Sceintific, Grand Island, NY, USA) supplemented with 10% charcoal-stripped serum (SH30068.03, Hyclone, Cytiva, Marlborough, MA, USA), penicillin (10 units/mL), and streptomycin (10 µg/mL) (designated as complete CSS media).

### 2.4. Immunoblot Analysis

For immunoblot analysis with estrogen treatment, cell lines (MCF-7, T-47D, or BT-474 cells) were seeded in 12-well plates in complete media. When cells reached ~90% confluency, complete media was aspirated; cells were washed with PBS once and were cultured in complete CSS media for 24 h. Cells were then treated with A-485 or GNE-049 for 24 h in complete CSS media. Estrogen (E2, 1 nM) was added 6 h before the addition of Passive Lysis Buffer (PLB) for cell lysis (5X PLB: 125 mM Tris, pH 7.8, 10 mM 1,2-CDTA, 10 mM DTT, 5 mg/mL bovine serum albumin, 5% (vol/vol) Triton X-100 and 50% (vol/vol) glycerol in ddH_2_O). Immunoblotting procedures were performed as described previously [58]. Briefly, samples were run on a 4–20% gradient polyacrylamide gel (XP04205BOX, Invitrogen, ThermoFisher Scientific, Carlsbad, CA, USA) and transferred to a Immobilon^®^-P polyvinylidene difluoride (PVDF) membrane (MilliporeSigma, Burlington, MA, USA). Membranes were blocked with the blocking buffer (1X TBST containing 5% non-fat milk) and then incubated overnight with primary antibodies diluted in the blocking buffer. Membranes for immunoblots with multiple probes were stripped using 0.2 M NaOH and incubated with the next primary antibody. Primary antibodies used include Estrogen Receptor-α (SC-8002, Santa Cruz Biotechnology, Dallas, TX, USA), c-MYC (Ab32072, Abcam, Cambridge, MA, USA), Cyclin D1 (Ab134175, Abcam), α-tubulin (T5168, MilliporeSigma, Burlington, MA, USA), GREB1 (28699-1-AP, ProteinTech, Rosemont, IL, USA), H3K27ac (8173, Cell Signaling Technology, Danvers, MA, USA), H3K18ac (9675, Cell Signaling Technology), H3K9ac (9649, Cell Signaling Technology), H3 (3638, Cell Signaling Technology), H2BK5ac (12799, Cell Signaling Technology), H2BK12ac (1755-1, Epitomics), H2B (12364BC, Cell Signaling Technology), p300 (SC-585, Santa Cruz Biotechnology), CBP (SC-7300, Santa Cruz Biotechnology), and GFP (SC-9996, Santa Cruz Biotechnology). Signal was detected using donkey anti-rabbit IgG (711-035-152, Jackson ImmunoResearch Laboratories, West Grove, PA, USA) and anti-mouse IgG HRP (7076, Cell Signaling Technology) using Immobilon Chemiluminescent HRP Substrate (WBKLS0500, MilliporeSigma, Burlington, MA, USA). Images were obtained on an x-ray film processor (SRX-101A, Konica Minolta Medical & Graphic, Inc., Tokyo, Japan) or an Amersham Imager 680 (Cytiva, Marlborough, MA, USA). Fuji Super RX-N X-ray films were used with the X-ray film processor. Densitometry quantification of the immunoblot bands was performed with ImageJ. For quantification, the probe of interest was normalized to the loading control for each sample and the fold change for each sample was then calculated using the DMSO- or the estrogen-treated sample as the reference. 

### 2.5. RT-qPCR of ER Target Genes 

MCF-7 cells were seeded in complete media in six well plates in duplicates for each treatment group. When cells reached ~90% confluency, media was aspirated; cells were washed once with PBS and were cultured in complete CSS media for 24 h. Cells were then treated with A-485 or GNE-049 for 24 h in complete CSS media. Estrogen (E2, 1 nM) was added 4 h before cell lysis and RNA extraction. RNA was extracted using the RNeasy (Qiagen, Germantown, MD, USA) kit according to manufacturer instructions. cDNA was generated according to manufacturer instructions using the SuperScript IV Reverse Transcriptase (Invitrogen, ThermoFisher Scientific, Carlsbad, CA, USA) kit or the Multiscribe reverse transcriptase kit (Applied Biosystems, ThermoFisher Scientific, Foster City, CA, USA). qPCR was performed on the Applied Biosystems Step One Plus Real Time PCR System according to the manufacturer instructions using the SYBR Green (Bimake, Houston, TX, USA) kit. qPCR data was analyzed using the ΔΔCt method with the Ct values of ACTB expression as the reference. Primer sequences are available in File S1. Representative data (out of two independent experiments, *n* = 2) is shown.

### 2.6. ERE Luciferase Assay 

MCF-7 cells were seeded in a 24-well plate in complete media. At 24 h after seeding, cells were transfected with a 3X ERE Tata Luc construct (Addgene 11354) at 300 ng and a plasmid encoding GFP (produced in-house) at 100 ng. Transfections were performed using Lipofectamine 3000 (Invitrogen, ThermoFisher Scientific, Carlsbad, CA, USA) according to manufacturer instructions. At 24 h after the transfection, cells were washed once with PBS and cultured in complete CSS media with DMSO, A-485, and GNE-049 at the indicated concentrations for 24 h. Estrogen was added 6 h before lysis in Passive Lysis Buffer. Luciferase readings were performed according to manufacturer instruction for the Luciferase Assay System (Promega, Madison, WI, USA) kit. The remaining lysate not used in the luciferase assay were utilized for immunoblotting using the protocol described above. 

### 2.7. Microarray and RNA-Seq Analysis 

MCF-7 cells were treated with A-485 at 3 µM for 24 h in complete media and RNA was extracted using the RNeasy kit (Qiagen). RNA was used for microarray analysis at the University of Florida Interdisciplinary Center for Biotechnology Research. RNAs were processed for microarray hybridization to the Affymetrix GeneChip Human Transcriptome Array 2.0. For data analysis, we performed differential expression (DE) analysis using R package limma to compare the DMSO group and the A-485 group. Limma utilizes linear model approach to identify differentially expressed genes and employs empirical Bayesian methods to stabilize the variance estimation. Differentially expressed genes with *p*-value < 0.05 and fold change (FC) ≥ 1.25 or ≤−1.25 were identified. Differentially expressed genes are shown in Table S2.

We used public gene expression datasets from MCF-7 cells based on the microarray technology (GEO GSE76200 [59]) and RNA-seq technology (GSE109957 [60]) to identify differentially expressed genes due to EP300 knockdown by siRNA or shRNA as above. Differentially expressed genes based on the RNA-seq data are available in Table S2. Differentially expressed genes based on the microarray data are available from a public dataset (GSE109957).

### 2.8. Immunofluorescence Microscopy 

MCF-7 cells were seeded on a glass coverslip placed in a 6 well plate in complete media. When cells reached ~90% confluency, cells were treated with the indicated compound for 48 h. After the drug incubation, cells were fixed with 4% paraformaldehyde, permeabilized with 0.2% Triton X-100 and incubated with a primary antibody for 90 min at room temperature. Primary antibodies against p300 (Santa Cruz Biotechnology, SC-585) and CBP (Cell Signaling Technologies, 7389) were used. Samples were then incubated with a proper secondary antibody for 30 min at room temperature and mounted on microscope slides (Fisher, 12-544-1) with VECTASHIELD mounting medium (Vector Laboratories, Burlingame, CA, USA) for imaging. The secondary antibodies goat anti-rabbit IgG Alexa, Fluor 488 (Life Technologies, A-11008) and goat anti-mouse IgG Alexa, Fluor-594 (Life Technologies, A-11005) were used. Images were captured using the iVision software and the Zeiss Axiophot microscope with a QImaging EXi Blue camera.

### 2.9. Chromatin Immunoprecipitation (ChIP), Library Preparation, Sequencing (ChIP-Seq), and ChIP-qPCR

MCF-7 cells were seeded in a 15-cm dish in complete media and grown to ~90% confluency. Once at the desired confluency, cells were incubated with A-485 at 3 µM for 24 h in complete media. ChIP was then performed as described previously [58]. Briefly, cells were fixed in 1% formaldehyde (final concentration) for 10 min; cells were lysed, and chromatin was sonicated to an average 200 bp. Immunoprecipitation was performed using an anti-H3K27ac antibody (8173, Cell Signaling Technology) and normal rabbit IgG (P120-101, Bethyl Laboratories, Montgomery, TX, USA). Input samples (1%) were also saved for library production. Immunoprecipitated complexes were washed, eluted from beads, treated with Proteinase K and crosslinks were reversed. DNA was purified using a PCR Cleanup Kit (28104, Qiagen). Two independent experiments (*n* = 2) were used for library production and sequencing. DNA libraries were produced according to manufacturer instructions using the NebNext Ultra II DNA Library Prep Kit for Illumina (New England Biolabs, Ipswich, MA, USA). Multiplex Oligos for Illumina (Index Primers Set 1 and Set 2) from New England Biolabs were used as index primers during library preparation. Paired-end sequencing of the DNA libraries was performed by Novogene Co. using the Illumina HiSeq PE150 platform.

For ChIP-qPCR, MCF-7 cells were cultured and treated with A-485 or DMSO; cells were fixed and subjected to ChIP as above. The recovered DNAs from ChIP were used for qPCR on an Applied Biosystems Step One Plus Real Time PCR System according to the manufacturer instructions using SYBR Green. qPCR Ct values were analyzed using the 1% input method and normalized to the DMSO control for two independent experiments (*n* = 2). Primer sequences are available File S1.

### 2.10. ChIP-Seq Data Analysis 

The quality of the sequencing reads was assessed by FastQC (https://www.bioinformatics.babraham.ac.uk/projects/fastqc/, accessed on 10 January 2018). The fastq reads were aligned to the Genome Reference Consortium Human Build 38 (hg38) using Bowtie2 [61]. In Bowtie2, the default parameters were used with the local alignment option, which does not require reads to align end-to-end, and the low-quality reads or adaptor reads are trimmed at one or both ends to optimize the alignment score. Duplicated reads were removed by SAMtools [62]. The alignment results of the biological replicates for the drug treatment groups were merged. Peak detection for samples treated with DMSO and A-485 were performed using HomeR [63], with corresponding input samples as background control. For peak detection, HomeR utilizes the Poisson model to obtain the significance level of the identified peaks. For each sample, the peak tags (i.e., number of sequencing reads within a peak region) were normalized as the counts per 10^7^ reads. We further denote the peak intensity as the log2-transformed normalized peak tags. We adopted the default parameter setting in HomeR to filter putative peaks: (i) Normalized tags of the putative peak should be at least 4-fold higher than the background control; (ii) the density at peaks should be at least 4-fold higher than the 10 kb surrounding regions; (iii) the peaks near the repetitive element region, which shows odd tag distribution, were removed. Peak annotation was also performed using HomeR to determine the genomic annotation of each peak, including promoter-TSS, exon, intron, intergenic regions. The promoter-TSS region is defined as the region between 1 kb upstream and 100 bp downstream of the TSS. Each peak was annotated to a gene whose TSS is closest to the peak. To compare the peak density at the promoter regions between A-485-treated sample and DMSO-treated control, we visualized the normalized coverage of ±2 kb extended regions of the peaks annotated with promoters-TSS. A paired student *t*-test was performed to examine the difference in normalized coverage between samples treated with A-485 and DMSO. HomeR was also used to identify super enhancers using a detailed procedure that has been previously described [63]. Briefly, adjacent peaks were merged as regions, and a super enhancer score of each region was determined by its normalized read counts subtracting the normalized reads of the input. After further normalizing against the highest score and against the number of putative enhancer regions, a monotone increasing curve was fitted where x-axis was the sorted region index and y-axis was the super enhancer score. Super enhancers were identified as regions past the point where the slope of the curve was greater than 1.

### 2.11. ChromHMM Analysis 

To infer and characterize the chromatin states of the identified peaks, we performed ChromHMM analysis [64], which utilizes a multivariate Hidden Markov Model (HMM) to decide the presence or absence of a chromatin state. We integrated six histone marks in ChIP datasets derived from the ER+ MCF-7 cell line including H3K27ac, H3K27me3, H3K36me3, H3K4me1, H3K4me3, H3K9ac, and H3K9me3, which are publicly available in GEO (GSE85158). ChromHMM model was trained using these chromatin marks and 18 chromatin states were identified according to the combinations of individual chromatin marks. In particular, we identified State 14 as an Active Enhancer, State 15 as Flanking TSS, and State 17 as Active TSS as described previously [65]. Data is displayed using boxplots (with their associated p values) and statistical significance was determined using the Wilcoxon Rank Sum Test.

### 2.12. Association of ChIP-Seq Data and Microarray Gene Expression Data 

We extracted the gene symbols that are downregulated by A-485 from the microarray data. We visualized the log normalized tags in ChIP-Seq peaks in samples treated with DMSO and A-485 of the downregulated genes via boxplot (with their associated p values). Statistical significance was determined using the Wilcoxon Rank Sum Test.

### 2.13. Cell Viability, Growth, and Colony Formation Assays 

MCF-7, T-47D, and BT-474 cells were seeded in a 96-well plate in complete media. At 24 h after seeding, cells were treated with an indicated compound for 96 h in complete media. After the drug incubation, cell viability was measured according to the manufacturer instructions for the CellTiter Glo (Promega) assay. Cell viability of samples treated with a CBP/p300 inhibitor was normalized to that of the DMSO control for three independent experiments (*n* = 3).

For in vitro cell growth assays, MCF-7 and BT-474 cells were seeded in a 24-well (MCF-7) or 12-well (BT-474) plate in complete media. At 24 h after seeding, cells were treated with an indicated compound for 8 days in complete media. Media and drugs were replaced every four days. After 8 days of drug incubation, cells were trypsinized and counted using the TC20 (Bio-Rad Laboratories, Hercules, CA, USA) automated cell counter. Cell numbers of samples treated with a CBP/p300 inhibitor were normalized to that of samples treated with DMSO for three independent experiments (*n* = 3). For long-term cell growth, MCF-7 cells were treated as above and cells were split, counted, and re-seeded in a 24-well plate. Cells were then allowed to grow in the absence of drugs for 9 days, after which they were counted as above. Cell number of samples treated with a CBP/p300 inhibitor was normalized to that of samples treated with DMSO for three independent experiments (*n* = 3).

For colony formation assay, MCF-7, T-47D and BT-474 cells were seeded in a 6-well plate in complete media. At 24 h after seeding, cells were treated with an indicated compound in complete media. Media and drugs were replaced every four days. Colonies were allowed to grow for 14 days (MCF-7) or 21 days (BT-474 and T-47D). Colonies were then fixed with 4% paraformaldehyde and stained with crystal violet. Colony area was measured with the ColonyArea ImageJ plugin. Colony area of wells with cells treated with A-485 or GNE-049 was normalized to that of DMSO-treated wells for three independent experiments (*n* = 3). Representative images of plates with colonies were taken using the ColonyArea ImageJ plugin [66].

### 2.14. Senescence Staining and Image Analysis 

MCF-7 cells were seeded in a 6-well plate in complete media. At 24 h after seeding, cells were treated with DMSO or A-485 (3 µM) for 8 days in complete media. Media and drugs were replaced every four days. After 8 days of drug incubation, cells were trypsinized and counted using the TC20 (Bio-Rad) automated cell counter. Five thousand cells were then re-seeded in a 96-well plate (in triplicate) for the DMSO and A-485 groups. At 24 h after seeding, cells were fixed and stained for β-galactosidase activity using the Senescence Green Detection Kit (C10850, Invitrogen, ThermoFisher Scientific, Carlsbad, CA, USA). Images of each technical replicate were taken using a Leica DMI 4000b microscope and staining intensity was measured using Cell Profiler. Briefly, images were uploaded to Cell Profiler and converted to gray scale. Cells were identified manually, and mean intensity of cells was measured using the MeasureObjectIntensity module. A total of 30 cells were measured for each group in the independent experiment (10 cells from each of the technical replicates). Mean intensity was then plotted for two independent experiments (*n* = 2) with each data point representing the mean intensity of 10 cells measured from each of the technical replicates for the two independent experiments (in total 60 cells in DMSO and 60 cells in A-485).

### 2.15. Statistical Analysis

The student’s *t*-test was used for studies with two experimental groups. ANOVA was used to analyze experiments with more than two groups. When ANOVA is used, the student’s *t*-test or Welch’s *t*-test was utilized as a post hoc test. If a different statistical test was utilized, it is noted in the methods section for that method. A *p* value of <0.05 was considered statistically significant. *, **, and *** denote *p* < 0.05, 0.005 and 0.0005, respectively. Quantitative data were presented as plots along with standard error of the mean (SEM) as error bars.

## 3. Results

### 3.1. Upregulation of CBP/p300 in BC Cell Lines and Tumors

CBP/p300 have a clear role in regulating the activity of pro-growth signaling pathways in BC and as such are emerging therapeutic targets [21,52,66]. However, how CBP/p300 promote BC growth remains largely unexplored. Based on the Cancer Dependency Map [54], BC cell lines appear highly dependent on p300 for survival (ranking as 5th most dependent cancer on p300, Figure S1A). BC ranked as the 2nd most dependent cancer on CBP, even though the median gene effect scores for CBP are not strongly negative. In validation of these findings, known CREBBP/EP300 dependent cancer types (i.e., PCa, lymphoma, myeloma, and leukemia [29,67]) all ranked highly for CREBBP/EP300 dependency versus other cancer types (Figure S1A). Interestingly, there appears to be a strong, positive correlation between CREBBP and EP300 expression in BC cell lines (Figure S1B). Similar findings were seen in primary breast tumors, including ER+ tumors, from the TCGA BRCA dataset (Figure S1C,D) [56]. Notably, both CREBBP and EP300 mRNA levels are increased in ER+ BC tumors compared to ER- tumors (Figure S1E). These observations suggest both CBP and p300 are promising therapeutic targets, especially in ER+ BC subtypes.

### 3.2. ER Is a Major Interaction Partner for Both CBP and p300 in Luminal BC

**Figure 1:**
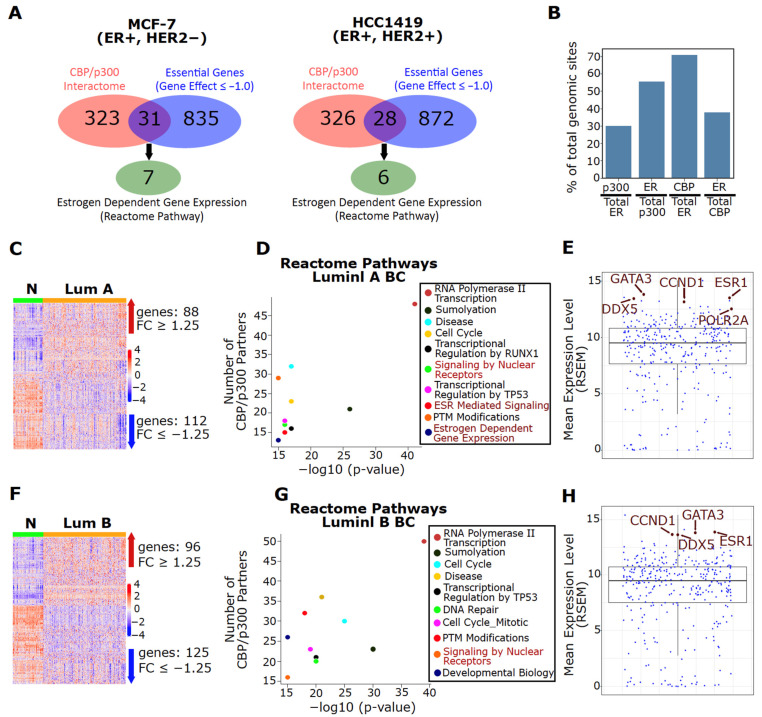
The CBP/p300 interactome is dysregulated in luminal BC and ER is a major interaction partner for both CBP and p300. (**A**) Few CBP/p300 binding partners are essential for ER+ BC growth. Essential CBP/p300 binding partners (gene effect score ≤ −1) were identified in ER+ MCF-7 and HCC1419 BC cells. (**B**) ER shares many chromatin-binding sites with p300 and CBP in MCF-7 cells. The shared binding sites are represented as a percentage of shared chromatin-binding sites of the two factors noted in each bar with respect to total binding sites of p300, CBP and ER (data from Zwart et al. [52] and Yi et al. [21]). (**C**,**F**) The CBP/p300 interactome is dysregulated in luminal A (panel **C**) and B (panel **F**) BC. Differentially expressed (q-value < 0.05, |FC| ≥ 1.25) CBP/p300 binding partners are visualized via heatmap for luminal A (**C**) and B (**F**) BC subtypes. Data are from TCGA [57]. (**D**,**G**) CBP/p300 binding partners involved in ER signaling are upregulated in luminal BC. CBP/p300-binding partners that are upregulated (|FC| ≥ 1.25, q-value < 0.05) in luminal A (panel **D**) and B (panel **G**) BC were analyzed for Reactome Pathway enrichment. The top statistically enriched pathways are shown and pathways in red are involved in ER signaling. (**E**,**H**) Partners involved in ER signaling are among the top 14 most highly expressed CBP/p300 partners in luminal A (panel **E**) and B (panel **H**) BC (data from TCGA). The mean expression of each CBP/p300-binding partner was plotted and gene symbols in brown are members of the Estrogen Dependent Gene Expression Reactome Pathway.

CBP/p300 are co-activators for numerous transcription factors and are known to interact with over 400 proteins [37]. We investigated which members of the CBP/p300 interactome are essential for ER+ BC growth and examined whether the ER pathway is the dominant CBP/p300-regulated pathway in ER+ BC (a list of binding partners in Table S1). Using the gene dependency scores from The Cancer Dependency Map, we determined which binding partners of CBP/p300 are essential for MCF-7 (luminal A) and HCC1419 (luminal B) (Gene effect score ≤ −1). Surprisingly, only 8.8% (31/354) and 7.9% (28/354) of the CBP/p300 interactome are essential for cell viability in MCF-7 and HCC149 cell lines, respectively (Figure 1A, Table S1). Approximately 22% of these essential binding partners (including ESR1, which encodes ER) are members of the Estrogen Dependent Gene Expression Reactome Pathway (Figure 1A). At the chromatin level, it is reported that ~56% and 38% of all p300 and CBP binding sites, respectively, are shared with ER in MCF-7 cells (Figure 1B, data from [21,52]). These results strongly imply that CBP/p300 function is skewed towards regulating ER signaling in ER+ BC. 

Next, we analyzed the expression profile of the CBP/p300 interactome based on TCGA tumor samples of luminal A and B BC subtypes. As expected of CBP/p300 as critical transcriptional co-activators, RNA polymerase II transcription is the top-ranked pathway among CBP/p300 binding partners that are upregulated in luminal breast tumors versus normal tissues (FC ≥ 1.25, q < 0.05). The upregulated CBP/p300 binding partners are also highly enriched for ER signaling pathways (marked in red) (Figure 1C,D, Table S1). Significantly, five of the 14 most highly expressed CBP/p300 binding partners in luminal A BC are members of the Estrogen Dependent Gene Expression Reactome Pathway (marked in brown) (Figure 1E). Importantly, ESR1 is one of the top 10 most highly expressed binding partners in luminal A tumors, as measured by mean expression level and mean rank (Figure 1E and Figure S2A). Similar results are seen in luminal B BC (Figure 1F–H, Table S1). Collectively, these bioinformatics analyses indicate that CBP/p300 are critical co-activators of ER in luminal BC.

### 3.3. CBP/p300 KAT (A-485) and BD (GNE-049) Inhibitors Attenuate Estrogen-Induced Gene Expression and ER Activity in ER+ BC Cells

**Figure 2:**
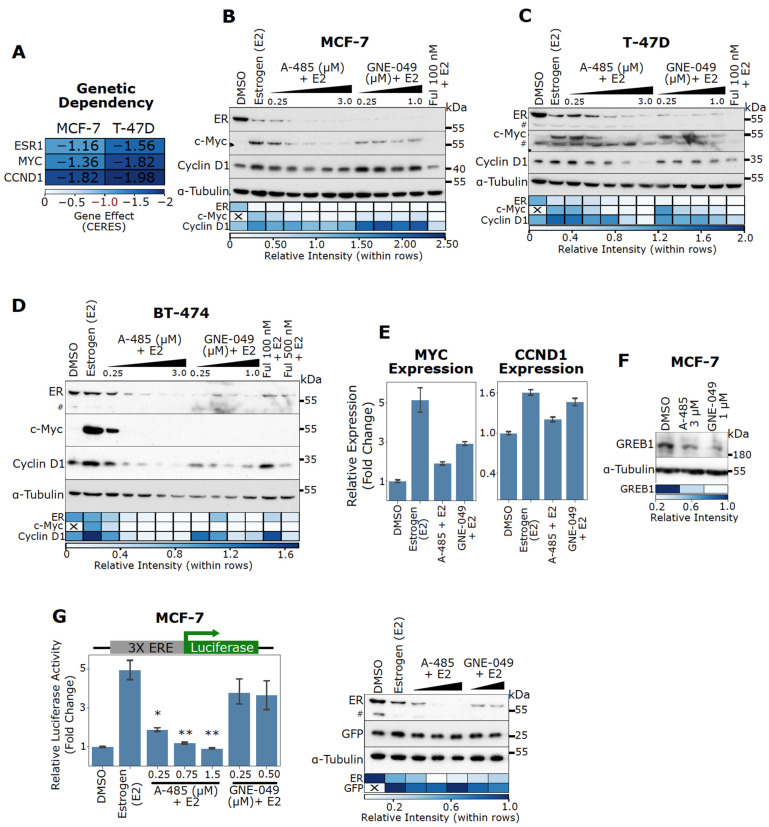
CBP/p300 KAT inhibitor A-485 and BD inhibitor GNE-049 downregulate ER protein levels and antagonize E2-stimulated activation of ER target genes MYC and CCND1. (**A**) ESR1, MYC, and CCND1 are essential in ER+ BC cells as determined by the Cancer Dependency Map. A Gene Effect of 0 is the median nonessential KO effect and -1 is the median essential KO effect. (**B**–**D**) CBP/p300 inhibitors potently reduce ER protein levels and antagonize E2-induced c-Myc expression in MCF-7 (**B**), T-47D (**C**), and BT-474 (**D**) cells. Cells were cultured in complete CSS medium, and then exposed to A-485 and GNE-049 for 24 h and E2 (1 nM) was added 6 h before cell lysis. ER, c-Myc and Cyclin D1 protein levels were assessed via immunoblotting. Densitometry quantification of the immunoblot bands are depicted below via heatmap. (**E**) Representative qPCR results for MYC and CCND1 expression in MCF-7 cells treated with E2 (1 nM), A-485 (750 nM), GNE-049 (250 nM) or their combination. Cells were cultured in complete CSS media and treated with CBP/p300 inhibitors for 24 h. E2 was added 4 h before lysis. Representative results of *n* = 2. Errors bars are SEM of two technical replicates. (**F**) A-485 and GNE-049 reduce GREB1 expression. MCF-7 cells were treated with A-485 and GNE-049 for 24 h in complete media and GREB1 protein levels were detected by immunoblotting. Densitometry quantification of the immunoblot bands are depicted below via heatmap. (**G**) A-485 potently blocks E2-activation of an ERE luciferase reporter. MCF-7 cells were transfected with a 3X ERE luciferase reporter plasmid and GFP. Cells in complete CSS media were exposed to varying doses of A-485 and GNE-049 for 24 h. E2 (1 nM) was added 6 h before cell lysis and luciferase activity was measured. A representative immunoblot is shown to the right for ER and GFP expression (representative of *n* = 3). Densitometry quantification of the immunoblot bands are depicted below via heatmap. Hashtags (#) denote non-specific band in immunoblots in panels C, D and G. In the bar graph in panel G, *: *p* < 0.05; **: *p* < 0.005 (Student’s *t*-test, *n* = 3).

In this study, the commonly studied ER+ BC cell lines of luminal A (MCF-7 and T-47D) and luminal B (BT-474) subtypes were utilized. Early studies identified MYC and CCND1 as critical ER target genes responsible for estrogen-induced cell cycle progression [4,5,6,7]. Indeed, MYC and CCND1 are essential genes for MCF-7 and T-47D cell viability based on CRISPR knockout data (Figure 2A). Although the gene effect data based on CRISPR for BT-474 are not available in the DepMap portal, high dependency on ESR1, MYC and CCND1 for cell viability of BT-474 are expected. ER, c-Myc, and Cyclin D1 are also known to promote tamoxifen resistance [15]. Notably, high MYC and CCND1 expression also correlates with poor relapse-free survival in BC patients treated with endocrine therapies (though it should be noted that the results for CCND1 are not as statistically significant as those for MYC, Figure S3A,B). Therefore, we analyzed the expression levels of ER, Myc and Cyclin D1 to assess the effects of CBP/p300 inhibitors on ER signaling. To directly assess the effects on ER signaling, cells were cultured in charcoal-stripped serum and then stimulated with estrogen (E2) in the absence or presence of a CBP/p300 inhibitor. As shown in Figure 2B–D, the CBP/p300 KAT inhibitor A-485 and BD inhibitor GNE-049 dose-dependently attenuate estrogen-induced c-Myc upregulation in MCF-7, T-47D, and BT-474 cells. A-485 and GNE-049 also strongly inhibit estrogen-induced Cyclin D1 expression in T-47D and BT-474 cells (Figure 2C,D). Strikingly, A-485 and GNE-049 potently downregulate ER protein levels in all three cell lines, indicating that CBP/p300 is essential for ER activity (Figure 2B–D). Of note, E2 induces marked ER downregulation in MCF-7 and T-47D cell lines, consistent with observations by others that E2 treatment induces proteosomal degradation of ER [68]. The SERD fulvestrant (Ful) is used as a positive control and similar results were seen (Figure 2B–D). 

In MCF-7 cells, A-485 (750 nM) and GNE-049 (250 nM) repress estrogen-induced mRNA expression of MYC (Figure 2E). In contrast, A-485 and GNE-049 have minimal effects on CCND1 expression at this concentration (Figure 2E), mimicking the results seen with immunoblotting (Figure 2B). This indicates MYC expression is highly sensitive to CBP/p300 inhibitors in MCF-7 cells. Of note, CCND1 expression did not seem to be perturbed by GNE-049 in MCF-7 cells (Figure 2B). A-485 and GNE-049 also reduce expression of GREB1, a canonical ER target gene in MCF-7 cells (Figure 2F). These results indicate that CBP/p300 inhibitors act through suppressing ER-mediated transcription. We further observed that A-485 inhibits ER transactivation activity using a luciferase reporter driven by three ERE motifs (Figure 2G). The co-transfected GFP accumulates at similar levels in the cell lysates used for the luciferase assay, indicating equal transfection efficiency (Figure 2G). ER downregulation was similarly observed in cells treated with A-485 or GNE-049 in the transfected cells (Figure 2G). Of note, GNE-049 did not reach statistical significance in this assay. These results collectively indicate that A-485 and GNE-049 inhibit ER-mediated transcription of clinically significant ER target genes.

### 3.4. CBP/p300 Are Critical for ER Signaling and Expression of Estrogen-Regulated Genes 

**Figure 3:**
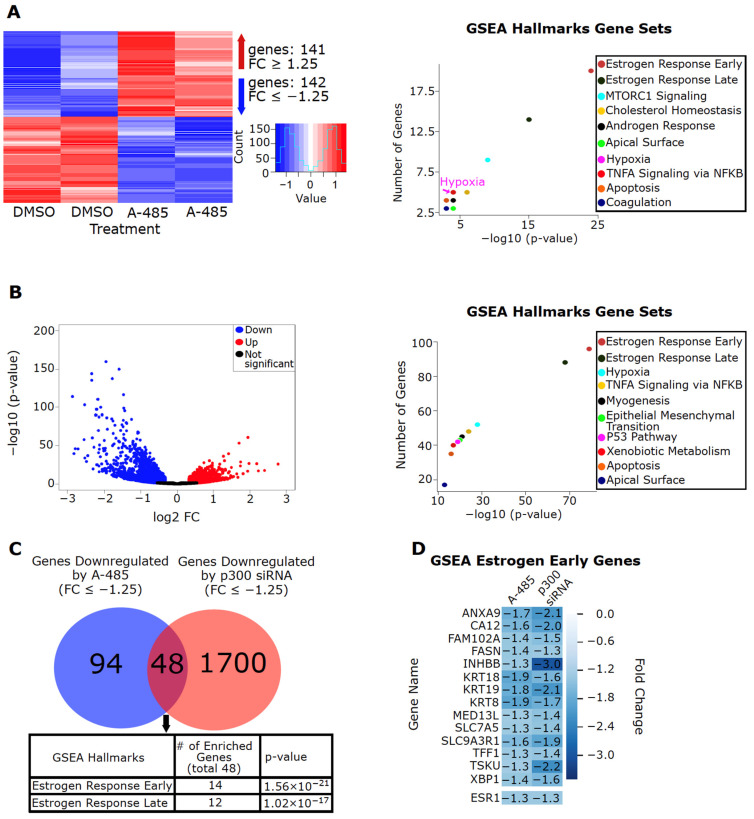
p300 is critical for the expression of estrogen-regulated genes in MCF-7 cells. (**A**) A-485 downregulates expression of estrogen-regulated genes in MCF-7 cells. Global gene expression in MCF-7 cells treated with A-485 (3 µM) for 24 h was analyzed using microarray. A-485 upregulates 141 genes (FC ≥ 1.25, *p* < 0.05) and downregulates 142 genes (FC ≤ −1.25, *p* < 0.05). The downregulated genes were analyzed for their involvement in biological pathways using GSEA Hallmarks Gene Sets. The top statistically enriched pathways are shown (right). (**B**) Genetic depletion of EP300 via siRNA downregulates expression of estrogen-regulated genes in MCF-7 cells. The log2 FC of all differentially expressed genes (*p* < 0.05) are shown. The downregulated genes were analyzed for their involvement in biological pathways using GSEA Hallmarks Gene Sets. The top statistically enriched pathways are shown (right). (**C**) CBP/p300 KAT inhibitor A-485 and EP300 genetic depletion impair ER signaling similarly. The genes repressed by A-485 in (**A**) were compared to the genes downregulated by EP300 genetic depletion (**B**). Genes downregulated by both were analyzed for their involvement in biological pathways using GSEA Hallmarks Gene Sets. The top statistically enriched pathways are shown below. (**D**) Members of the GSEA Estrogen Early Hallmark Gene Set downregulated by both A-485 and EP300 genetic depletion are shown with their respective fold changes.

To assess global effects of CBP/p300 inhibition by A-485, we performed a microarray analysis on MCF-7 cells treated with DMSO or A-485. Gene-set enrichment analysis (GSEA) using the Molecular Signatures Database (MSigDB) of the A-485 downregulated genes (FC ≤ −1.25, *p* < 0.05) based on the microarray data show that genes involved in the Estrogen Response Early and Estrogen Response Late Hallmark Gene Sets are the top two enriched pathways (Figure 3A, Table S2). Consistent with immunoblotting data shown above (Figure 2), ESR1 expression was reduced in the microarray data (Figure 3D). The reduced ESR1 expression was confirmed with RT-qPCR, along with FASN and TFF1, two genes in the Estrogen Response Early Gene Set (Figure S4A). Downregulation of EP300 expression with siRNA in MCF-7 cells also results in global repression of genes involved in the Estrogen Response Early and Estrogen Response Late Gene Sets (Figure 3B, data from GSE109957 [60]). Similar results were seen with an additional public dataset with EP300 genetic knockdown in MCF-7 cells (Figure S4B, data from GSE76200 [59], Table S2). 

Comparison of the genes downregulated by A-485 and EP300 genetic knockdown reveals that the commonly downregulated genes are enriched for estrogen-regulated genes (Figure 3C). Figure 3D shows commonly downregulated genes (including ESR1) by A-485 or EP300 knockdown in the Estrogen Response Early Gene Set. These results confirm that CBP/p300 globally regulates ER signaling and demonstrate that the ER pathway is the primary target of CBP/p300 inhibitors in ER+ BC cells.

To investigate if the genes downregulated by A-485 are clinically significant and if pharmacological CBP/p300 inhibition could potentially restore ER+ tumors to a more “normal-like” gene expression pattern, we compared the genes downregulated by A-485 (Figure 3A) and EP300 knockdown (Figure 3B) to genes known to be upregulated in luminal A and B tumors versus normal tissues (Figure S5A,B). Indeed, 36% (51/142) and 37% (53/142) of genes downregulated by A-485 in MCF-7 cells are upregulated in luminal A and B BC, respectively (Figure S5A). Furthermore, the Estrogen Response Early and Estrogen Response Late Hallmark Gene Sets are the top two enriched pathways for the overlapping genes (Figure S5A). Likewise, 31.8% (556/1748) and 30.2% (528/1748) of genes downregulated by EP300 knockdown in MCF-7 cells are upregulated in luminal A and B BC, respectively (Figure S5B), and again the Estrogen Response Early and Estrogen Response Late Hallmark Gene Sets are the top two enriched pathways for the overlapping genes (Figure S5B). Together these results imply that CBP/p300 inhibition could effectively suppress ER signaling and thus restore ER+ tumors to a more “normal-like” gene expression pattern for genes downstream of ER signaling.

### 3.5. Effects of A-485 and GNE-049 on Histone Acetylation

**Figure 4:**
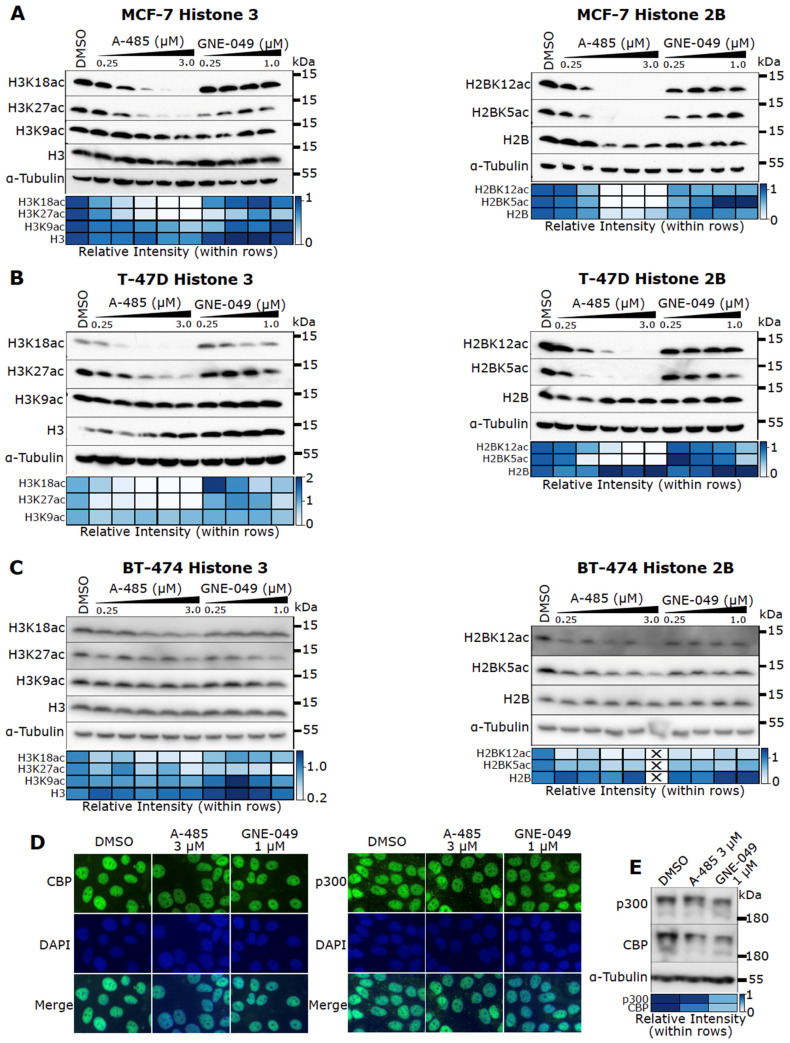
Inhibition of specific histone acetylation by A-485 and GNE-049 in ER+ BC cells. (**A**–**C**) MCF-7 (**A**), T-47D (**B**), and BT-474 (**C**) cells were treated with A-485 and GNE-049 for 24 h before cell lysis. Acetylation at specific H3 (left) and H2B sites (right) were probed via immunoblotting. Densitometry quantification of the immunoblot bands is depicted below via heatmap. (**D**) A-485 and GNE-049 do not alter CBP/p300 subcellular location. MCF-7 cells were treated with A-485 and GNE-049 for 48 h and subjected to immunofluorescence microscopy with antibodies against p300 and CBP. Nuclei are counterstained with DAPI. Representative images are shown. (**E**) MCF-7 cells were treated with A-485 and GNE-049 for 24 h before cell lysis. p300 and CBP protein levels were probed via immunoblotting. Densitometry quantification of the immunoblot bands is depicted below via heatmap.

The data presented above indicate that pharmacological inhibition of CBP/p300 represents a potent means to ablate ER signaling. Next, we sought to determine the mechanism by which CBP/p300 inhibitors block ER activity. Consistent with the findings that CBP/p300 specifically catalyze H3K27 and H3K18 acetylation [39], A-485 markedly reduces H3K18ac and H3K27ac in all cell lines we tested. In contrast, it has little effect on H3K9ac (Figure 4A–C). A-485 also potently downregulates H2BK5ac and H2BK12ac (Figure 4A-C), in agreement with previous findings that these modifications are mediated by CBP/p300 [38]. The BD inhibitor GNE-049, by contrast, only decreases H3K27ac without significantly affecting acetylation of other H3 or H2B sites in ER+ BC cells (Figure 4A–C).

Notably, A-485 and GNE-049 neither obviously affect CBP/p300 subcellular localization, as determined through immunofluorescence microscopy (Figure 4D), nor impact overall CBP/p300 protein level (Figure 4E). These results support the notion that these inhibitors act through inhibiting the KAT activity or the BD function of CBP/p300. 

### 3.6. A-485 Preferentially Suppresses H3K27ac at Active Enhancers

CBP/p300 binding is enriched at enhancers [69,70] and CBP/p300-catalyzed H3K27ac is an established marker of transcriptionally active enhancers [43,71,72,73]. It was previously reported that GNE-049 downregulates H3K27ac at enhancers to a greater degree than that at promoter regions [40]. These findings led Raisner et al. to conclude that the CBP/p300 BD has an “enhancer-biased” role in H3K27 acetylation [40]. Nevertheless, it is also observed that CBP/p300-mediated acetylation in promoters may directly impact transcriptional outputs [74]. However, how pharmacological inhibition of CBP/p300’s catalytic activity affects genome-wide H3K27ac has not been reported. Therefore, we conducted H3K27ac ChIP-seq experiments to assess genome-wide impact of A-485.

**Figure 5:**
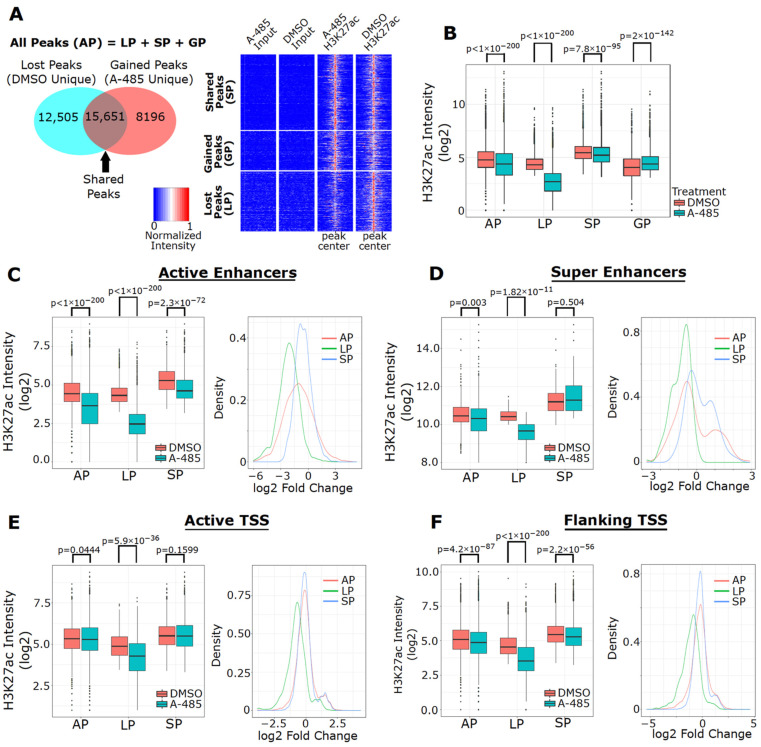
A-485 preferentially suppresses H3K27ac at enhancers. (**A**) Venn diagram of the number of H3K27ac peaks detected in four peak types (All Peak (AP), Lost Peak (LP), Gained Peak (GP) and Shared Peak (SP)) in MCF-7 cells treated with A-485 (3 µM) for 24 h (left). Heatmaps depicting normalized H3K27ac peak intensity (at peak center ± 3 kb) in Lost, Gained and Shared peaks are shown (right). (**B**) Boxplots of H3K27ac peak intensity for DMSO and A-485 in each of the peak types. Boxplots of H3K27ac peak intensity in MCF-7 cells treated with A-485 or DMSO (left) and fold-changes of H3K27ac (right) at active enhancers (**C**), super enhancers (**D**), active TSSs, (**E**) and flanking TSSs, (**F**) in each of the peak types.

Consistent with immunoblotting data (Figure 4), A-485 reduces overall H3K27ac peak intensity in MCF-7 cells (Figure 5A,B). In analyzing our ChIP-seq dataset, we divided the H3K27ac peaks into four groups (peak types): All Peaks (AP, all detected peaks), Lost Peaks (LP, DMSO unique peaks), Shared Peaks (SP, peaks detected in both DMSO and A-485), and Gained Peaks (GP, A-485 unique peaks) (Figure 5A). In total, 36,352 H3K27ac peaks are detected, among which the number of LPs and SPs is similar, while there are fewer GPs (~8000). A-485 markedly reduces H3K27ac peak intensity in LPs but only has a small effect on SPs (Figure 5B). We then utilized ChromHMM to discover chromatin-states that may define functional regulatory elements such as enhancers and promoters, based on the presence or absence of specific histone modification marks [64,65]. We chose six different histone marks (H3K4me3, H3K27ac, H3K4m1, H3K27me3, H3K9me3, and H3K36me3) from available datasets derived from MCF-7 cells to identify potential epigenomic regulatory regions that controls gene expression. Different combinations of these histone marks may define active and repressed enhancers and promoters. Our analysis generated 18 different chromatin states with a different combination of these histone marks, which can be used to define active enhancers and promoters (Figure S6A). We then annotated these states to the appropriate functional regions according to Ernst et al. [65] and further classified them based on their distance relative to transcription start sites (TSSs) (Figure S6B). Such computational annotations define State 14 as active enhancers, State 15 as flanking TSSs and State 17 as active TSSs (Figure S6A). These three chromatin states are the most enriched states in our dataset; in contrast, chromatin states with a high probability of containing repressive marks (e.g., H3K27me3 and H3K9me3) are not well-represented in our dataset (Figure S6C), suggesting that H3K27ac is largely associated with actively transcribed genes in MCF-7 cells.

Remarkably, H3K27ac peak intensity at active enhancers is highly reduced by A-485 (Figure 5C). Similar results were observed for H3K27ac peak intensity at super enhancers defined by the HomeR program (Figure 5D). Consistent with the results from cells treated with GNE-049, A-485 did not significantly affect H3K27ac peak intensity at active TSSs (i.e., promoters), although reduced H3K27ac was apparent at the TSSs of a subset of genes (Figure 5E). Similar results were seen when analyzing the flanking TSSs (Figure 5F). An independent analysis of H3K27ac peak intensity at TSSs using HomeR led to similar results (Figure S6D). Based on these findings, we conclude that inhibition of the catalytic activity of CBP/p300 impairs H3K27ac preferentially on active enhancers, with relatively limited effects on TSSs.

### 3.7. A-485 Inhibits H3K27ac at the Enhancers of Downregulated ER Target Genes

**Figure 6:**
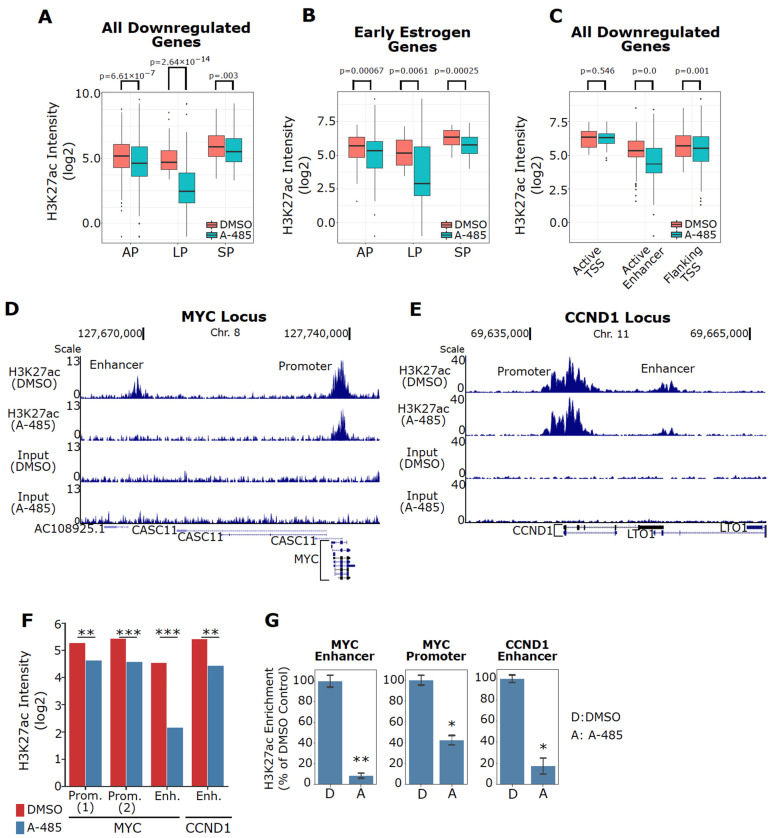
Specific inhibition of H3K27ac at ER target genes by A-485. (**A**) A-485 reduces H3K27ac intensity at the genes downregulated by A-485 in MCF-7 cells. (**B**) A-485 specifically reduces H3K27ac levels at estrogen-regulated genes that are repressed by A-485. The estrogen-regulated genes are from the members of the GSEA Estrogen Early Hallmark Gene Set. (**C**) A-485 reduces H3K27ac at active enhancers associated with transcriptionally downregulated genes identified in the microarray dataset (Figure 4A) in MCF-7 cells. (**D**,**E**) A UCSC genome browser track for the MYC (**D**) or CCND1 (**E**) locus. A-485 reduces H3K27ac at the MYC promoter and an ER-responsive upstream enhancer (**D**), while inhibiting H3K27ac at a CCND1 ER-responsive downstream enhancer (**E**) in MCF-7 cells. (**F**) Fold changes of H3K27ac intensity of ChIP-seq peaks associated with the MYC promoter (Promoter Peak 1 and Peak 2), MYC Enhancer, and CCND1 enhancer. (**G**) Validation of A-485-mediated inhibition of H3K27ac at the MYC promoter, MYC enhancer and CCND1 enhancer in MCF-7 cells, as measured by ChIP-qPCR (*n* = 2). *: *p* < 0.05; **: *p* < 0.005; *** *p* < 0.0005 (Poisson regression model by HomeR in panel **F** and Student’s *t*-test in panel **G**).

We demonstrated that A-485 specifically inhibits expression of estrogen-regulated genes (Figure 3A). To examine if reduced H3K27ac correlates with decreased gene expression by A-485, we interrogated our microarray gene expression data along with our H3K27ac ChIP-seq dataset. This analysis revealed that genes transcriptionally downregulated by A-485 based on our microarray data exhibit decreased overall H3K27ac peak intensity in our ChIP-seq data with potent reduction in H3K27ac intensity at Lost Peaks (Figure 6A). Similar results are seen at estrogen-regulated genes downregulated by A-485 (Figure 6B). The reduction of H3K27ac peak intensity at enhancers of the transcriptionally downregulated genes is obvious, in contrast to little effects at TSSs of these genes (Figure 6C). Of note, although in most cases A-485 does not affect H3K27ac at TSSs, some downregulated genes show reduced H3K27ac at their TSSs (e.g., INHBB and CA12 in GSEA Early Estrogen Genes, Figure S7A,B). This indicates that A-485 can also suppress promoter acetylation to repress gene expression. Additionally, A-485 treatment resulted in mRNA upregulation and increased H3K27ac peak intensity for a subset of genes (e.g., PPDPF and GDF15, Figure S7C,D). This suggests that inhibition of CBP/p300 catalytic function does not uniformly reduce global H3K27ac, possibly due to chromatin dynamics during the A-485 treatment period (see Discussion).

To further understand how A-485 impacts the chromatin states of key ER target genes underlying oncogenesis, we focused on the MYC and CCND1 loci because they contain defined ER-responsive enhancers (i.e., directly bound by ER) that are also occupied by p300 to control their expression in MCF-7 cells. Notably, A-485 reduces H3K27ac peak intensity at the MYC promoter as well as an upstream region that overlaps with the MYC enhancer [5] (Figure 6D,F). A-485 also reduces H3K27ac at a downstream CCND1 ER-responsive enhancer [75] (Figure 6E,F). Using ChIP-qPCR, we confirmed reduced H3K27ac at these regions (Figure 6G). These results collectively demonstrate that A-485 acts by suppressing H3K27ac at ER target genes, thereby downregulating their expression.

Since our findings indicate A-485 suppresses transcription through reduced enhancer acetylation, we next investigated whether the enhancers having reduced H3K27ac due to A-485 treatment are clinically significant through comparison of genes whose enhancers have reduced H3K27ac to upregulated genes in ER+ tumors (Figure S8). This analysis indicates that 27.7% (190/687) and 26.9% (185/687) of genes associated with enhancers with reduced H3K27ac in our ChIP-seq data are also known to be upregulated in luminal A and luminal B BC, respectively (Figure S8A). The Estrogen Response Early and Estrogen Response Late Hallmark Gene Sets are the top two enriched pathways for the overlapping genes (Figure S8A). These results suggest that A-485 could potentially inhibit transcription of highly expressed genes in luminal BC tumors through a reduction of H3K27ac associated with these genes. 

### 3.8. Pharmacological Inhibition of CBP/p300 Suppresses Cell Growth and Induces Senescence 

**Figure 7:**
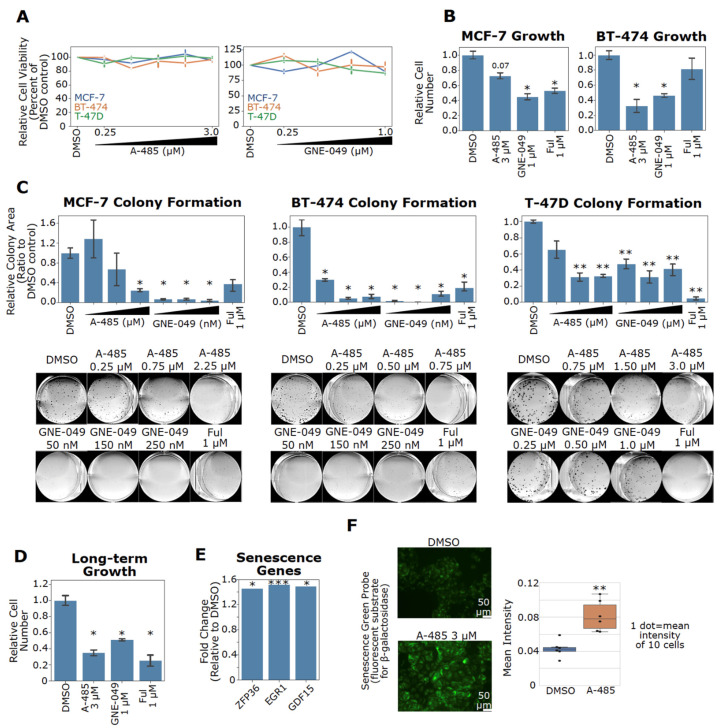
CBP/p300 inhibitors A-485 and GNE-049 suppress cell growth and induce senescence of ER+ BC cells. (**A**) CBP/p300 inhibitors do not acutely reduce cell viability in ER+ BC cells. MCF-7, T-47D, and BT-474 cells were treated with A-485 and GNE-049 at the indicated concentrations for 96 h. Cell viability was measured with Cell-TiterGlo. (**B**) CBP/p300 inhibitors block cell proliferation in ER+ BC cell lines. MCF-7 and BT-474 cells were treated with the indicated concentrations of A-485 and GNE-049 for 8 days. Cell numbers were then counted and the relative cell numbers are shown (normalized to the DMSO control, *n* = 3). (**C**) CBP/p300 inhibitors potently suppress colony formation in ER+ BC cell lines. MCF-7, BT-474 and T-47D cells were treated with the indicated concentrations of A-485 and GNE-049. Colonies were allowed to grow for between 2–3 weeks. Colony area was measured by ImageJ plugin ColonyArea and normalized to the DMSO control (*n* = 3). Representative colony images for each cell line are shown below. (**D**) Long-term CBP/p300 inhibition retards cell growth after drug washout. MCF-7 cells were treated as in (**B**) for 8 days. Cells were then counted, re-seeded and allowed to grow for 9 days in the absence of drugs. The relative growth of drug treated cells vs DMSO control from three independent experiments (*n* = 3) is plotted. (**E**) A-485 upregulates senescence-associated genes in MCF-7 cells. Data are from the microarray experiment. Fold changes of these genes are plotted. (**F**) A-485 induces senescence in MCF-7 cells. MCF-7 cells were treated with A-485 for 8 days. Cells were then counted and re-seeded in 96 well plates. At 24 h after reseeding, cells were stained for β-galactosidase activity and mean intensity of staining was quantified. Representative images of two independent experiments (*n* = 2) are shown. In the bar graph in panel G, *: *p* < 0.05; **: *p* < 0.005; ***: *p* < 0.0005 (Student’s *t*-test).

The potent downregulation of ER, c-Myc and Cyclin D1 by A-485 and GNE-049 in ER+ BC cells is expected to impair their growth and proliferation. In short-term treatment, A-485 and GNE-049 did not obviously reduce cell viability, indicating that these compounds do not acutely induce cell death (Figure 7A). Nonetheless, both GNE-049 and A-485 blocked cell proliferation in ER+ BC cells (Figure 7B). To assess long-term effects of these CBP/p300 inhibitors, we used clonogenic cell growth assays. Both compounds exhibited profound suppression of cell growth in all ER+ BC cell lines tested (Figure 7C). Long-term A-485 treatment is known to induce senescence in other cancer models [76]. Therefore, we investigated whether CBP/p300 inhibitors have a long-term effect on ER+ BC growth. Indeed, MCF-7 cells treated with A-485, GNE-049 and fulvestrant show marked long-term growth inhibition after drug washout (Figure 7D). A-485 upregulated expression of genes involved in senescence (e.g., ZFP36, EGR1, and GDF15 [77,78,79]) in our microarray data of A-485-treated MCF-7 cells (Figure 7E). Long-term A-485 treatment also increased β-galactosidase activity, a commonly used senescence marker, in MCF-7 cells (Figure 7F). These results suggest that pharmacological inhibition of CBP/p300 blocks cell proliferation through inducing senescence in ER+ BC.

## 4. Discussion

ER+ BC accounts for more than two-thirds of all BC cases and most BC deaths occur in ER+ BC. Tamoxifen, the standard-of-care therapy for ER+ BC, is met with resistance in 20–30% of patients [10,11,13]. Additional therapeutics, such as CDK4/6 inhibitors, are available for ER+ BC treatment. However, ~20% of patients exhibit de novo resistance to CDK4/6 inhibitors, and all patients ultimately develop resistance [19]. Thus, there is an urgent need for novel therapies to address these clinical concerns and to expand the repertoire of possible drug combinations that can be used in the clinic. 

ER-mediated oncogene expression is a major driver of tumor growth in ER+ BC [1,2,4,5,6,7]. Mechanistically, ER recruits co-activators to activate its target genes and these co-activators represent potential therapeutic targets [1,20,21,22,23,24,25]. CBP and p300 are two critical ER co-activators. In this study, we demonstrate that CBP/p300 are upregulated in ER+ BC. In analyzing the interactome of CBP/p300, we found that their binding partners involved in ER signaling are essential for ER+ BC growth and that these binding partners are upregulated in luminal A and B BCs. Significantly, the ER chromatin-binding sites largely overlap with those of CBP/p300 in the genome, indicating that ER recruits CBP/p300 to a majority of its binding sites [21,52]. Together, these results strongly imply that CBP/p300 is skewed towards regulating ER signaling versus other CBP/p300-regulated pathways in ER+ BC. Consequently, pharmacological inhibition of CBP/p300 likely strongly suppresses the growth of ER+ BC. 

Indeed, we observed that the CBP/p300 KAT inhibitor A-485 and their BD inhibitor GNE-049 impair the expression of specific oncogenes (e.g., ESR1, MYC, and CCND1) and A-485 globally suppresses ER signaling. Both A-485 treatment and EP300 genetic knockdown similarly repress estrogen-regulated genes, indicating that ER signaling ablation is an on-target effect of A-485. Mechanistically, our data show that A-485 suppresses H3K27ac at the enhancers of ER target genes, resulting in the downregulation of ER target gene expression. For example, A-485 potently reduces H3K27ac at the ER-responsive MYC and CCND1 enhancers in MCF-7 cells (Figure 6). Inhibition of these and other oncogenes likely underlies growth inhibition of ER+ BC cells by these inhibitors. 

Of note, some regions of the genome display increased H3K27ac on A-485 treatment, which appears counterintuitive to A-485’s role as a KAT inhibitor. Although precisely why this occurs is unknown, we can speculate that A-485 treatment might render such genomic sites less susceptible to H3K27ac turnover, possibly due to altered HDAC recruitment. As a result, H3K27ac might be elevated at such sites in A-485 treated cells compared to cells exposed to DMSO. In this context, it is worth noting that treatment with the HDAC inhibitor MS-275 can decrease H3K27ac at many genomic sites instead of globally increasing acetylation as expected [80]. These results indicate that targeting epigenetic modifying enzymes does not uniformly affect all genomic sites. Further studies will be required to understand how chromatin dynamics impacts local/global effects of inhibitors targeting histone modifying enzymes, including CBP/p300.

Our findings show that CBP/p300 represent attractive therapeutic targets in ER+ BC and that specific inhibitors of CBP/p300 may be applicable for treating patients with ER+ BC. As upregulation of ESR1, MYC, and CCND1 is implicated in treatment resistance to endocrine therapies [15], downregulation of these oncogenes by CBP/p300 inhibitors suggests that these inhibitors may be useful in tumors that no longer respond to available therapies. Unfortunately, some CBP/p300 inhibitors, including A-485 and GNE-049, display poor drug properties in vivo. Improving the pharmaceutical properties of these compounds could facilitate clinical translation of pharmacological CBP/p300 inhibition. Additional medicinal chemistry optimization may lead to better drug candidates. For example, GNE-781 is a structural analog of GNE-049 that maintains inhibitory potency and selectivity for the CBP/p300 BD but is less-brain penetrant than GNE-049 [35]. This may make GNE-781 a safer alternative for future in vivo studies. 

## 5. Conclusions

In summary, our studies established the mechanism of action and phenotypic effects of the CBP/p300 KAT inhibitor A-485 in ER+ BC. A-485 potently inhibits ER target gene expression through downregulation of H3K27ac primarily at enhancers, resulting in inhibition of ER+ BC cell growth. Our findings provide a rationale for further preclinical and potential clinical studies of CBP/p300 inhibitors for treating ER+ BC.

## Data Availability

Microarray and ChIP-sequencing datasets generated in the study are available in the Gene Expression Omnibus (NCBI GEO). Microarray data (accession #GSE172175) and ChIP-seq data (accession #GSE172174).

## Supplementary Materials

The following are available online at https://www.mdpi.com/article/10.3390/cancers13112799/s1. Figure S1: CBP/p300 are positively correlated in BC cell lines/tumors and are upregulated in ER+ BC tumors, Figure S2: ESR1 is among the top 10 most highly expressed CBP/p300 partners in luminal BC, as measured by mean rank, Figure S3: MYC and CCND1 expression negatively correlate with relapse-free survival of patients treated with endocrine therapy, Figure S4: A-485 and EP300 genetic knockdown commonly repress ER target genes in MCF-7 cells, Figure S5: A-485 and EP300 KD reduce expression of genes that are upregulated in luminal BC, Figure S6: ChromHMM Analysis in MCF-7 cells, Figure S7: A-485 reduces H3K27ac at ER target genes but increases H3K27ac at a subset of genes, Figure S8: A-485 reduces H3K27ac at active enhancers associated with genes that are upregulated in luminal BC, File S1: list of primers used in qPCR, File S2: Raw western blot images.
