# Supplementary material for: Pharmacological Inhibition of CBP/p300 Blocks Estrogen Receptor Alpha (ERα) Function through Suppressing Enhancer H3K27 Acetylation in Luminal Breast Cancer
Source: Cancers (Basel). 2021 Jun 4;13(11):2799. doi: 10.3390/cancers13112799 (PMC8200112; doi:10.3390/cancers13112799)
Supplement: Supplementary file 1 [file cancers-13-02799-s001.zip › File S1.pdf]

RT-qPCR Primers

MYC qPCR

F: AGCGACTCTGAGGAGGAACA

R: CTCTGACCTTTTGCCAGGAG

CCND1 qPCR

F: CCCTCGGTGTCCTACTTCAA

R: AGGAAGCGGTCCAGGTAGTT

ESR1 qPCR

F: TGGAGATCTTCGACATGCTG

R: TCCAGAGACTTCAGGGTGCT

FASN qPCR

F: CACAGGGACAACCTGGAGTT

R: ACTCCACAGGTGGGAACAAG

TFF1 qPCR

F: CACCATGGAGAACAAGGTGA

R: TGACACCAGGAAAACCACAA

Actin qPCR

F: GCTCCTCCTGAGCGCAAGTACTC

R: GTGGACAGCGAGGCCAGGAT

ChIP Primers

MYC enhancer

F: CATCCAATAAACCTTCCTACCTGA

R: TGGCAGGTGTCCTAGAGCAT

MYC promoter

F: CTCTGGAACAGGCAGACACA

R: TGGATACCTTCCACCCAGAC

CCND1 enhancer

F: CAGTTTGTCTTCCCGGGTTA

R: TCATCCAGAGCAAACAGCAG
