# Supplementary material for: Pharmacological Inhibition of CBP/p300 Blocks Estrogen Receptor Alpha (ERα) Function through Suppressing Enhancer H3K27 Acetylation in Luminal Breast Cancer
Source: Cancers (Basel). 2021 Jun 4;13(11):2799. doi: 10.3390/cancers13112799 (PMC8200112; doi:10.3390/cancers13112799)
Supplement: Supplementary file 1 [file cancers-13-02799-s001.zip › File S2.pdf]

MCF-7 Raw Blots: Figure 2B

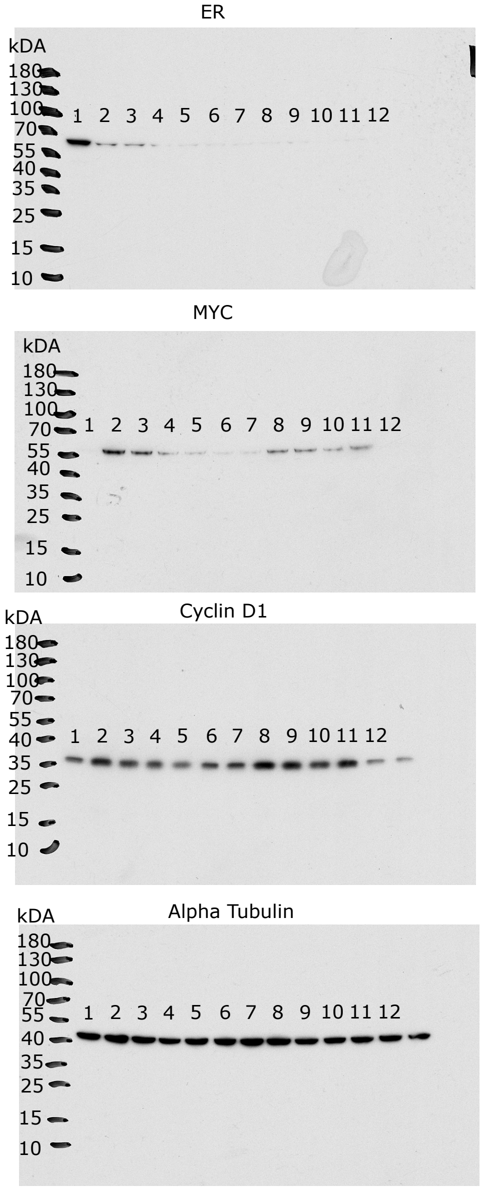

Key for all blots:
1. DMSO
2. Estrogen E2 1 nM
3. E2 + A-485 .25 μM
4. E2 + A-485 .75 μM
5. E2 + A-485 1.5 μM
6. E2 + A-485 2.25 μM
7. E2 + A-485 3 μM
8. E2 + GNE-049 .25 μM
9. E2 + GNE-049 .50 μM
10.E2+ GNE-049 .75 μM
11. E2 + GNE-049 1 μM
12. E2 + Fulvestrant 100 nM

T-47D Raw Blots: Figure 2C

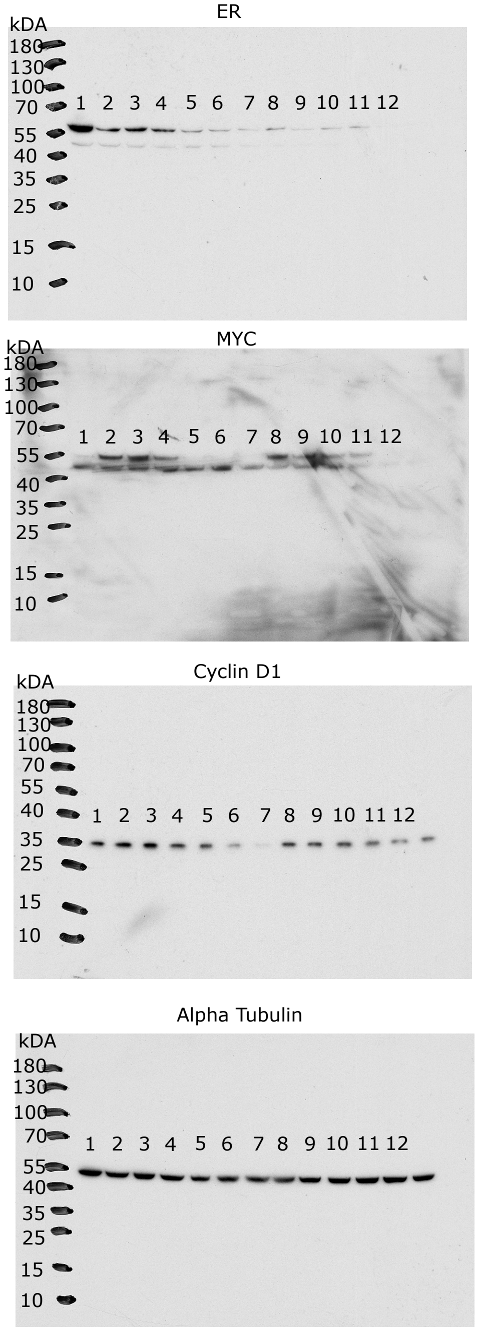

BT-474 Raw Blots: Figure 2D

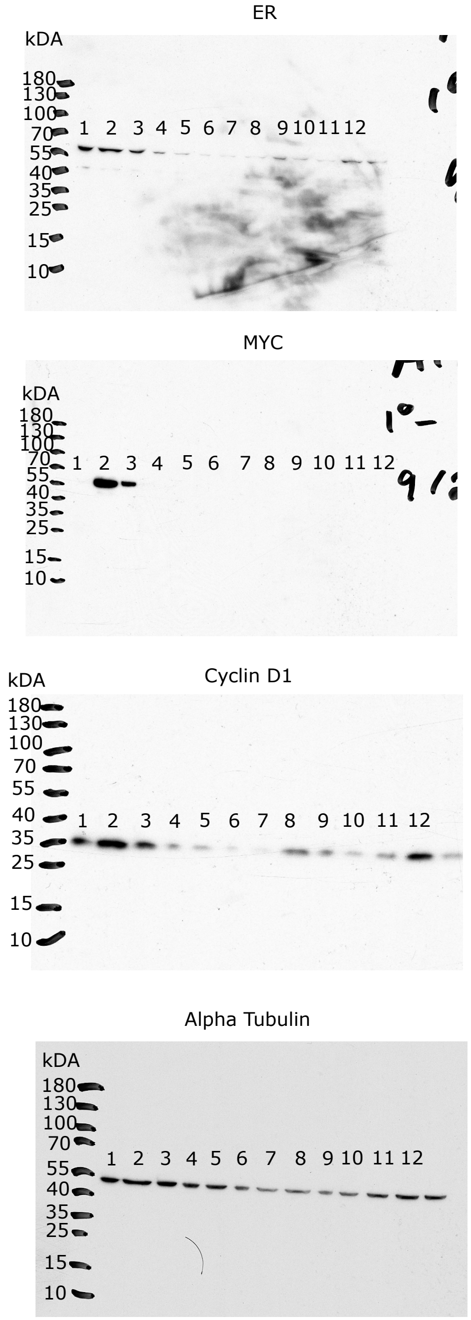

MCF-7 ERE Blot: Figure 2G

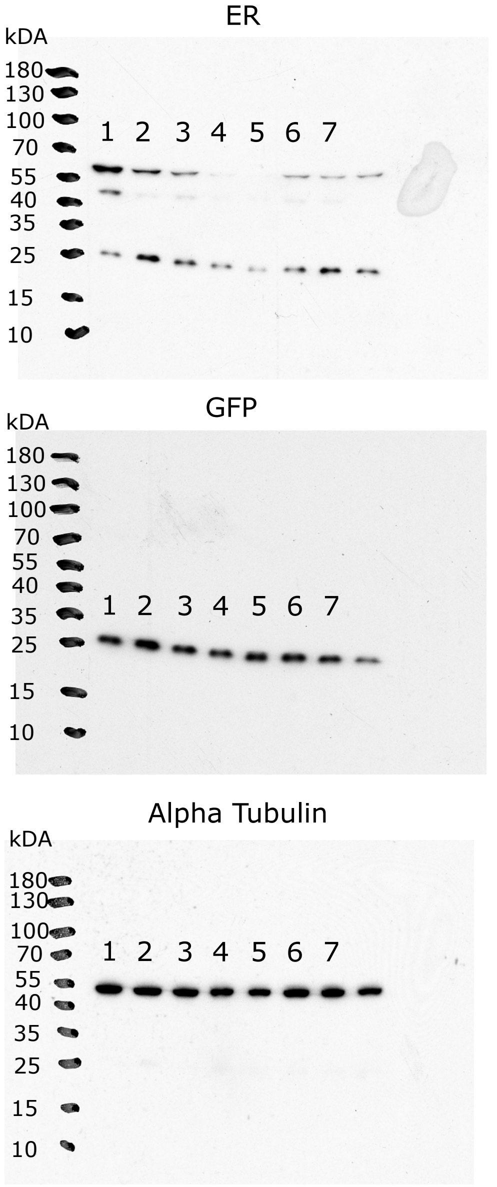

Key:
1. DMSO
2. Estrogen E2 1 nM
3. E2 + A-485 .25 μM
4. E2 + A-485 .75 μM
5. E2 + A-485 1.5 μM
6. E2 + GNE-049 .25 μM
7. E2 + GNE-049 .50 μM

MCF-7 GREB1 Blot: Figure 2G

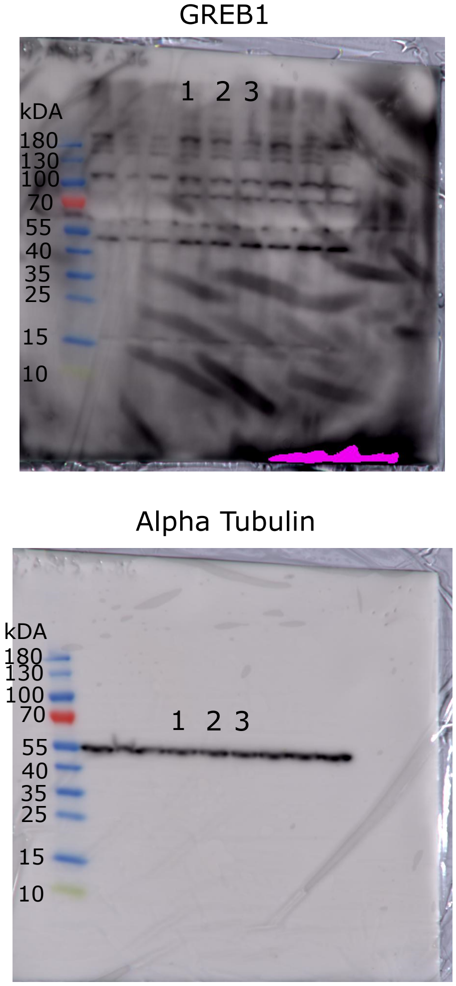

Key:
1. DMSO
2. A-485 3 μM
3. GNE-049 1 μM

MCF-7 H3ac Raw Blots:
Corresponds to Figure 4A

H3K18ac

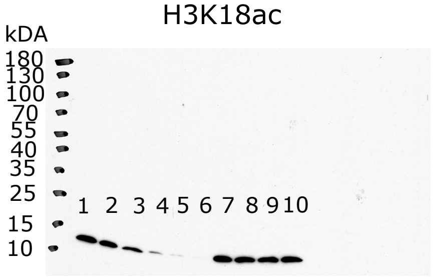

H3K27ac

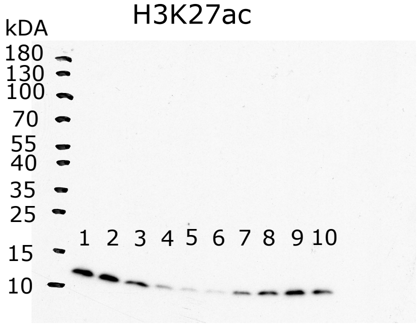

H3K9ac

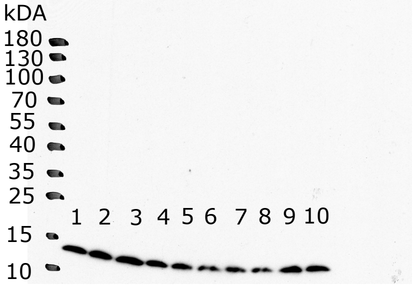

H3

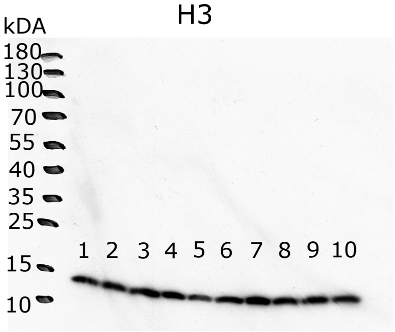

Alpha Tubulin

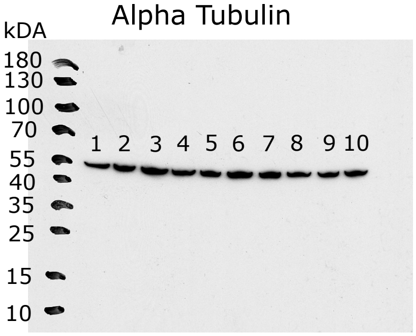

MCF-7 H2Bac Raw Blots:
Corresponds to Figure 4A

H2BK12ac

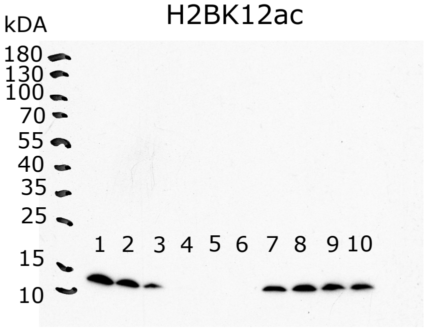

H2BK5ac

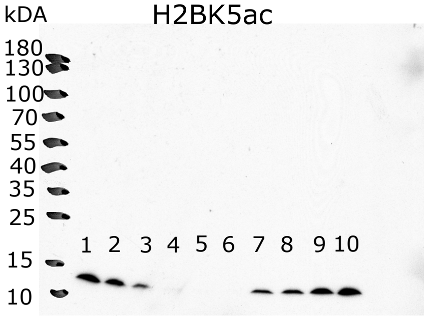

H2B

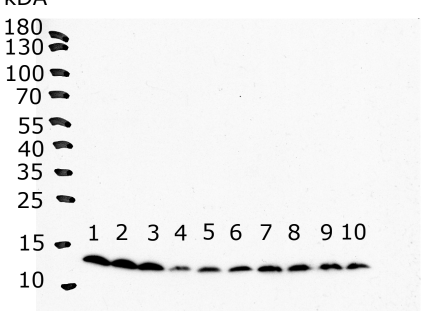

Alpha Tubulin

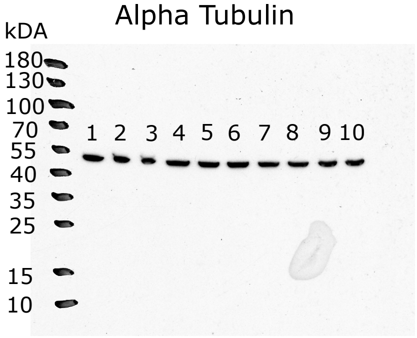

Key for all blots:
1. DMSO
2. A-485 .25 μM
3. A-485 .75 μM
4. A-485 1.5 μM
5. A-485 2.25 μM
6. A-485 3 μM
7. GNE-049 .25 μM
8. GNE-049 .50 μM
9. GNE-049 .75 μM
10. GNE-049 1 μM

T-47D H3ac Raw Blots: Figure 4B

H3K18ac

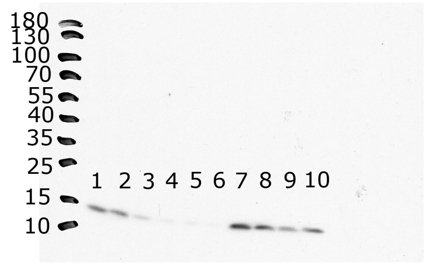

H3K27ac

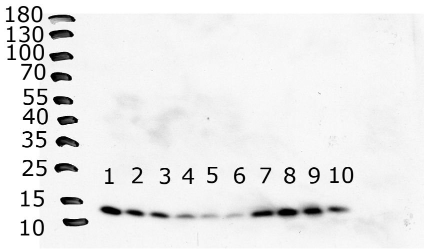

H3K9ac

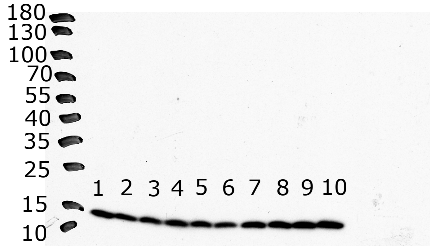

H3

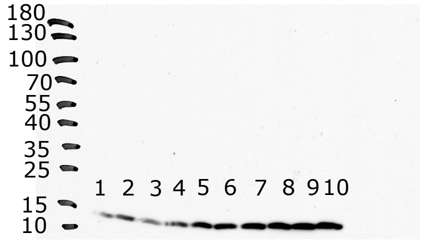

Alpha Tubulin

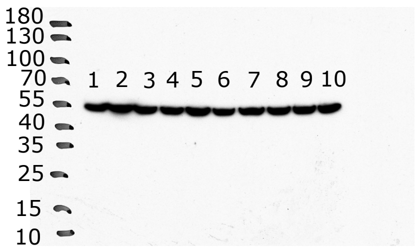

T-47D H2Bac Raw Blots: Figure 4B

H2BK12ac

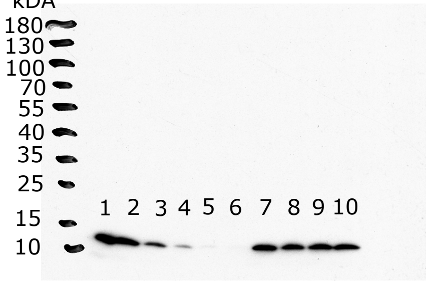

H2BK5ac

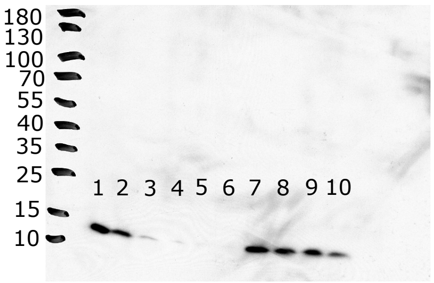

H2B

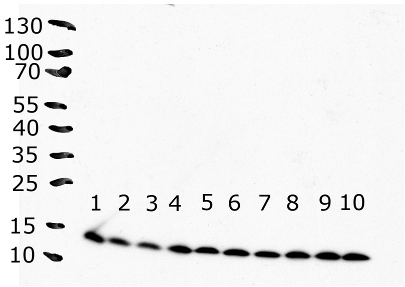

Alpha Tubulin

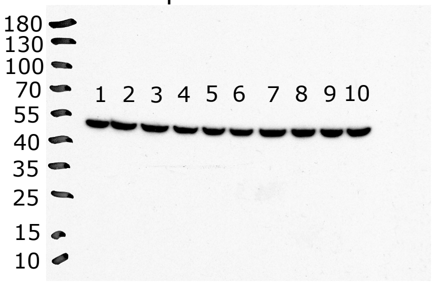

Key for all blots:
1. DMSO
2. A-485 .25 μM
3. A-485 .75 μM
4. A-485 1.5 μM
5. A-485 2.25 μM
6. A-485 3 μM
7. GNE-049 .25 μM
8. GNE-049 .50 μM
9. GNE-049 .75 μM
10. GNE-049 1 μM

BT-474 H3ac Raw Blots:
Figure 4C

H3K18ac

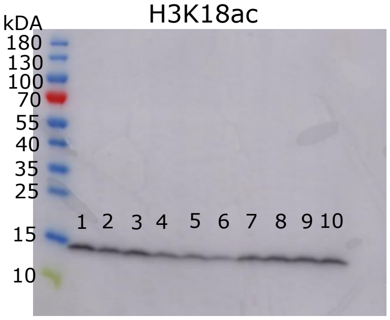

H3K27ac

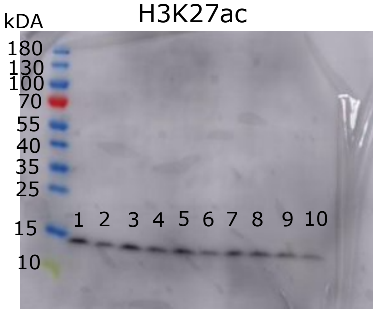

H3K9ac

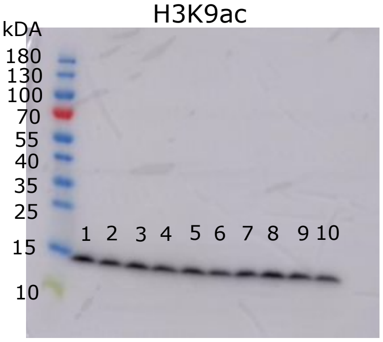

H3

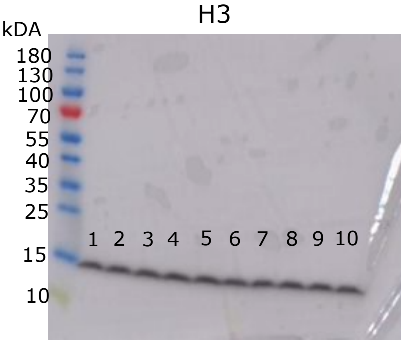

Alpha Tubulin

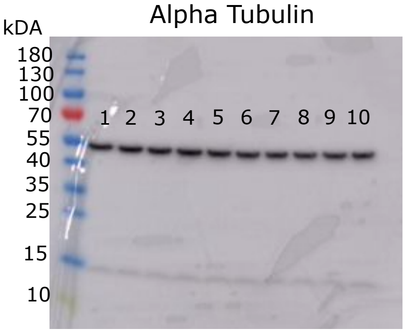

BT-474 H2Bac Raw Blots:
Figure 4C

H2BK12ac

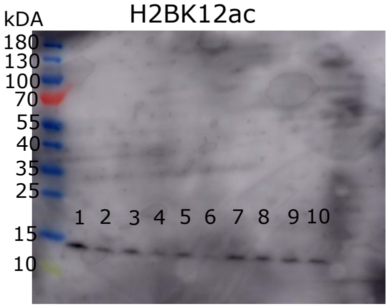

H2BK5ac

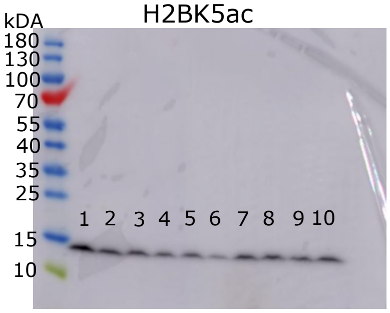

H2B

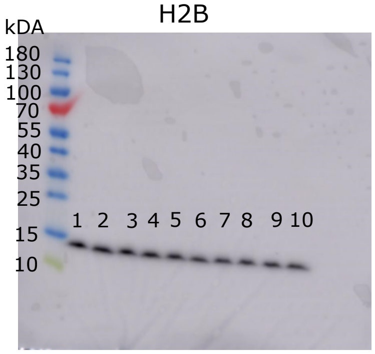

Alpha Tubulin

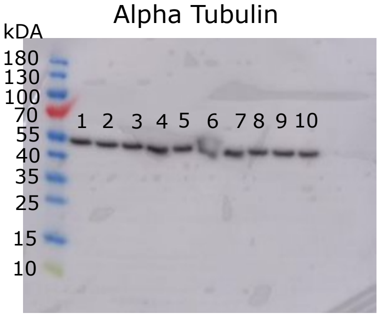

Key for BT-474 blots:
1. DMSO
2. A-485 .25 μM
3. A-485 .75 μM
4. A-485 1.5 μM
5. A-485 2.25 μM
6. A-485 3 μM
7. GNE-049 .25 μM
8. GNE-049 .50 μM
9. GNE-049 .75 μM
10. GNE-049 1 μM

MCF-7 CBP/p300 Raw Blots:
Figure 4E

p300

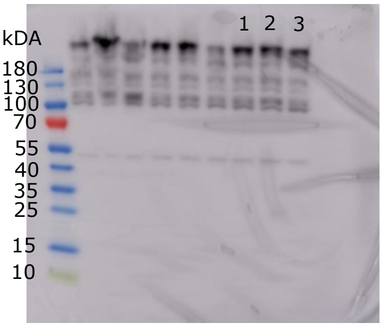

CBP

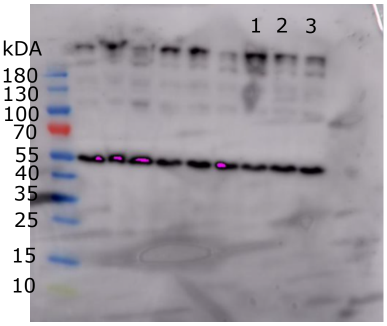

Alpha Tubulin

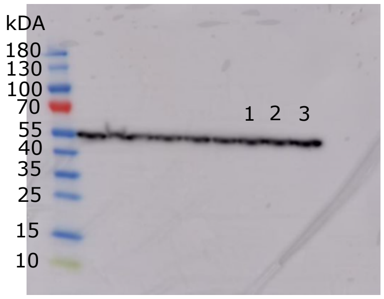

Key for MCF-7 blot:
1. DMSO
2. A-485 3.0 μM
3. GNE-049 1 μM
