# Supplementary material for: Pharmacological Inhibition of CBP/p300 Blocks Estrogen Receptor Alpha (ERα) Function through Suppressing Enhancer H3K27 Acetylation in Luminal Breast Cancer
Source: Cancers (Basel). 2021 Jun 4;13(11):2799. doi: 10.3390/cancers13112799 (PMC8200112; doi:10.3390/cancers13112799)
Supplement: Supplementary file 1 [file cancers-13-02799-s001.zip › Supplementary Figures.pdf]

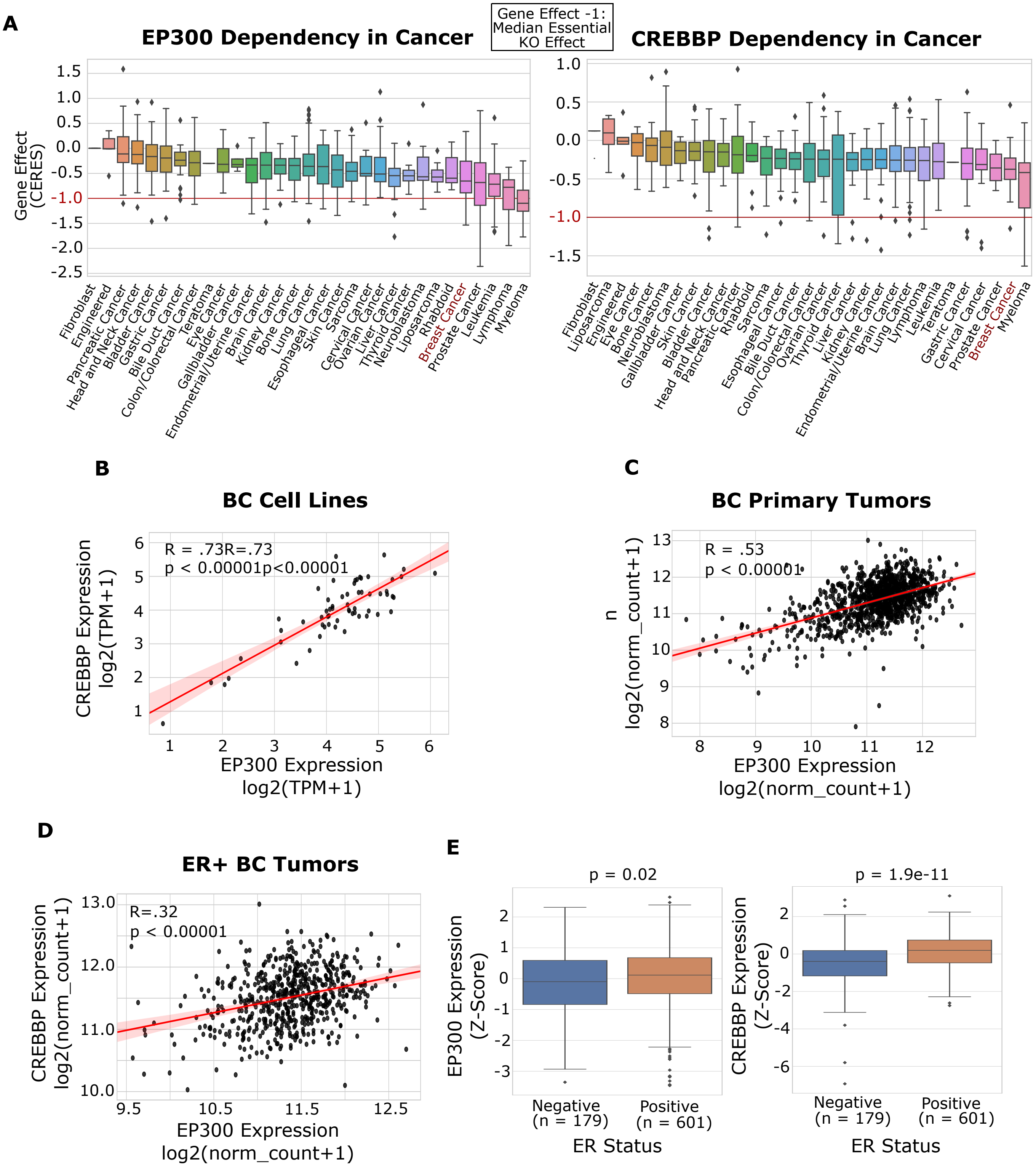
A
EP300 Dependency in Cancer
Gene Effect -1: Median Essential KO Effect
CREBBP Dependency in Cancer
Gene Effect (CERES)
1.5
1.0
0.5
0.0
-0.5
-1.0
-1.5
-2.0
-2.5
Fibroblast
Engineered
Pancreatic Cancer
Head and Neck Cancer
Bladder Cancer
Gastric Cancer
Bile Duct Cancer
Colon/Colorectal Cancer
Teratoma
Eye Cancer
Gallbladder Cancer
Endometrial/Uterine Cancer
Brain Cancer
Kidney Cancer
Bone Cancer
Lung Cancer
Esophageal Cancer
Skin Cancer
Sarcoma
Cervical Cancer
Ovarian Cancer
Liver Cancer
Thyroid Cancer
Neuroblastoma
Liposarcoma
Rhabdoid
Breast Cancer
Prostate Cancer
Leukemia
Lymphoma
Myeloma
Fibroblast
Liposarcoma
Engineered
Eye Cancer
Bone Cancer
Neuroblastoma
Gallbladder Cancer
Skin Cancer
Bladder Cancer
Head and Neck Cancer
Pancreatic Cancer
Rhabdoid
Sarcoma
Esophageal Cancer
Bile Duct Cancer
Colon/Colorectal Cancer
Ovarian Cancer
Thyroid Cancer
Liver Cancer
Endometrial/Uterine Cancer
Kidney Cancer
Brain Cancer
Lung Cancer
Lymphoma
Leukemia
Teratoma
Gastric Cancer
Cervical Cancer
Prostate Cancer
Breast Cancer
Myeloma
B
BC Cell Lines
R = .73R=.73
p < 0.00001p<0.00001
CREBBP Expression log2(TPM+1)
EP300 Expression log2(TPM+1)
C
BC Primary Tumors
R = .53
p < 0.00001
n
log2(norm_count+1)
EP300 Expression log2(norm_count+1)
D
ER+ BC Tumors
R=.32
p < 0.00001
CREBBP Expression log2(norm_count+1)
EP300 Expression log2(norm_count+1)
E
p = 0.02
p = 1.9e-11
EP300 Expression (Z-Score)
CREBBP Expression (Z-Score)
Negative (n = 179)
Positive (n = 601)
ER Status

Figure S1. CBP/p300 are positively correlated in BC cell lines/tumors and are upregulated in ER+ BC tumors

(A) Genetic dependency on EP300 (left) and CREBBP (right) in cancer cell lines. A Gene Effect of -1 represents the median essential knockout (KO) effect. Data from Cancer Dependency Map.

(B) EP300 and CREBBP expression are positively correlated in BC cell lines. EP300 and CREBBP mRNA expression values were obtained from the Cancer Dependency Map for BC cell lines. EP300 and CREBBP expression levels in the BC cell lines were plotted via scatterplot and the Pearson correlation coefficient (R) with its associated p-value is displayed.

(C) EP300 and CREBBP expression are positively correlated in BC primary tumors. EP300 and CREBBP mRNA expression values were obtained for primary BC tumors in the TCGA BRCA dataset using the Xena database. EP300 and CREBBP expression levels in the samples were plotted via scatterplot and the Pearson correlation coefficient (R) with its associated p-value is displayed.

(D) EP300 and CREBBP expression are positively correlated in primary ER+ BC tumors. EP300 and CREBBP mRNA expression values were obtained for primary ER+ BC tumors in the TCGA BRCA dataset using the Xena database. EP300 and CREBBP expression levels in the samples were plotted via scatterplot and the Pearson correlation coefficient (R) with its associated p-value is displayed.

(E) EP300 and CREBBP expression are upregulated in ER+ BC. EP300 and CREBBP mRNA expression values were obtained for primary BC tumors in the TCGA BRCA dataset and samples were filtered to analyze ER+ BC samples versus ER- BC samples using the Xena database.

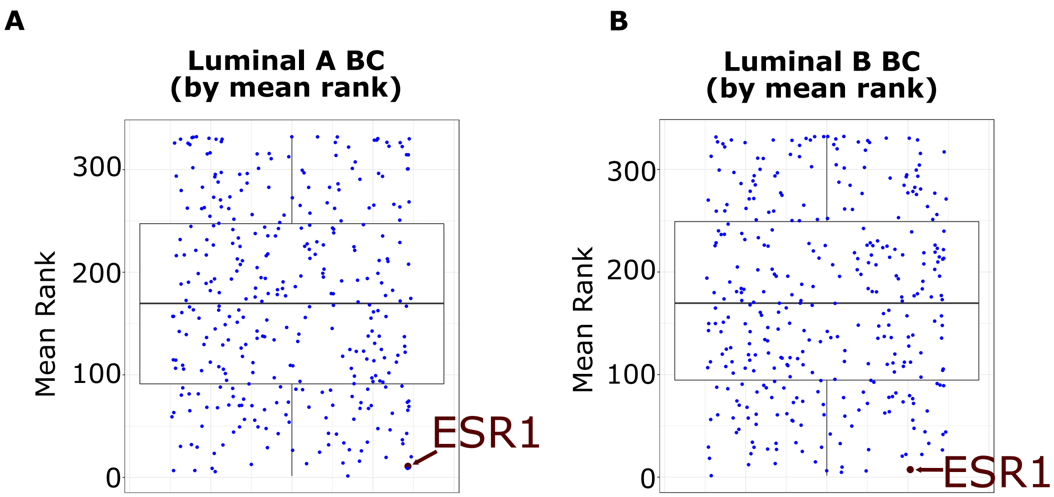

Figure S2. ESR1 is among the top 10 most highly expressed CBP/p300 partners in luminal BC, as measured by mean rank.

(A) ER is one of the top 10 most highly expressed CBP/p300 partners in luminal A BC (data from TCGA). The expression of each CBP/p300 interaction partner was ranked 1-354 (total 354 partners, with 1 having the highest mRNA expression value and 354 having the lowest) within each tumor sample. The mean rank of each CBP/p300 partner was then determined across samples and mean rank was plotted.
(B) ER is one of the top 10 most highly expressed CBP/p300 partners in luminal B BC (data from TCGA). The ranking of each CBP/p300 interaction partner and plotting were done as in (A).

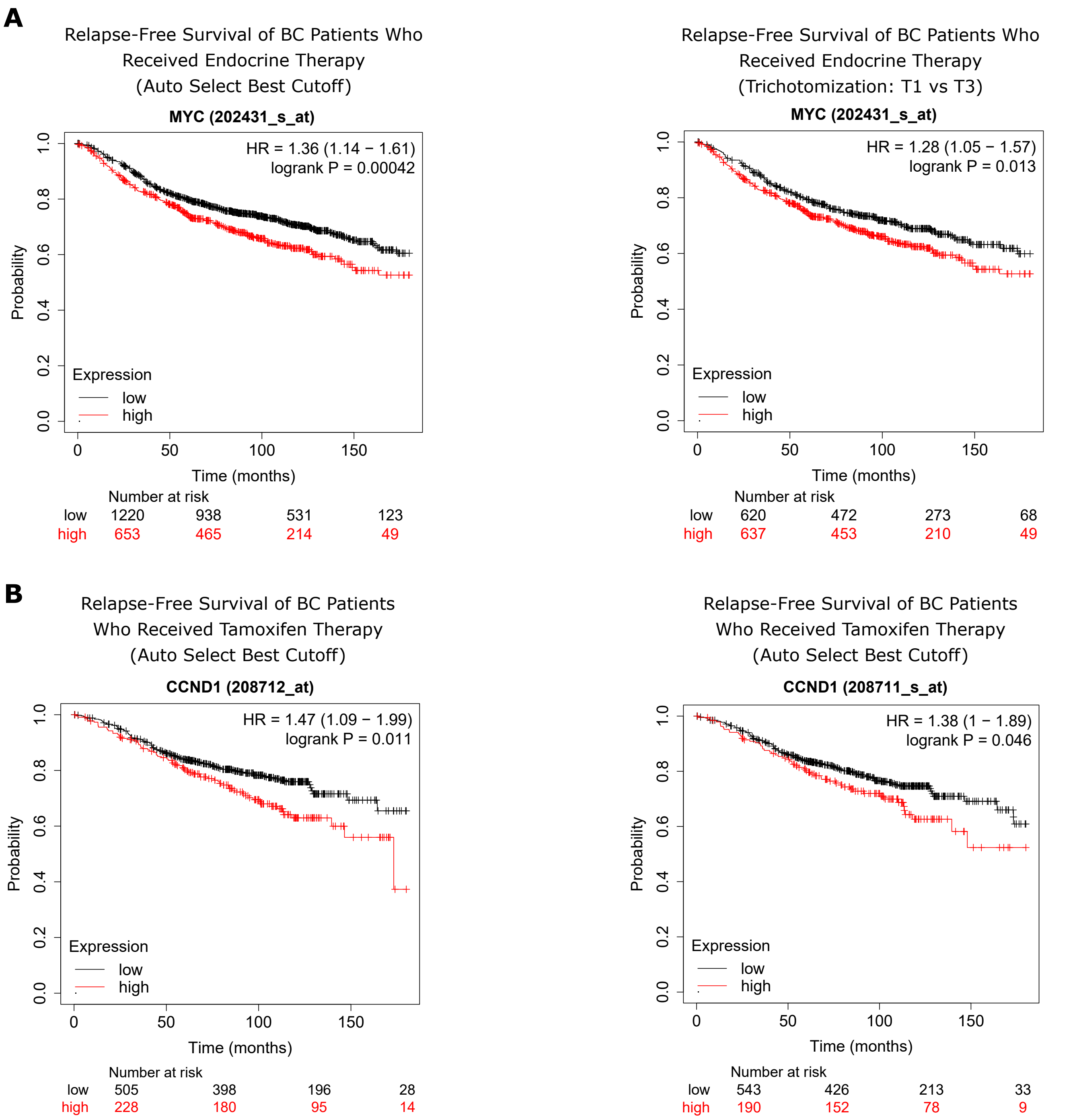

Figure S3: MYC and CCND1 expression negatively correlate with relapse-free survival of patients treated with endocrine therapy

(A) High MYC expression negatively correlates with relapse-free survival in patients treated with endocrine therapy, using two cutoff methods (auto:left and trichotomization:right). Data and plot from KM Plotter (kmplot.com)

(B) High CCND1 expression negatively correlates with relapse-free survival in patients treated with tamoxifen therapy. Plots for two different CCND1 probes are shown (left and right). Data and plot from KM Plotter (kmplot.com)

A

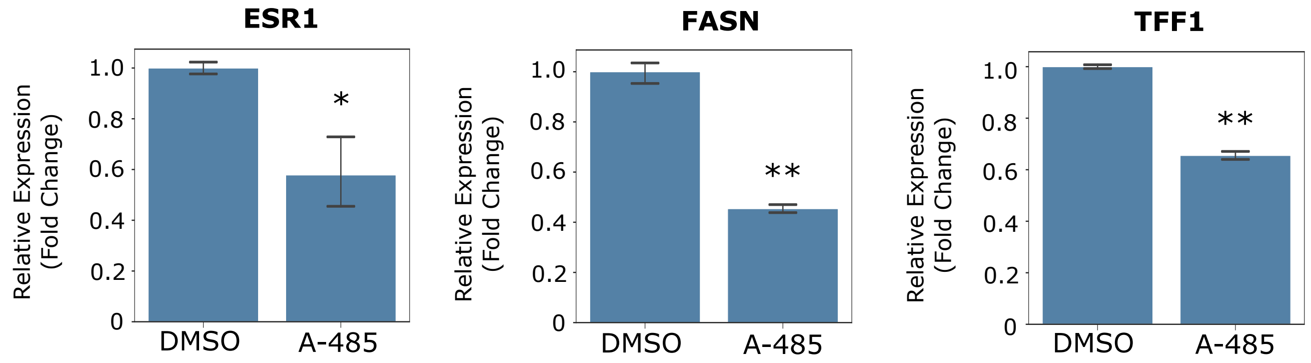

B

EP300 shRNA1

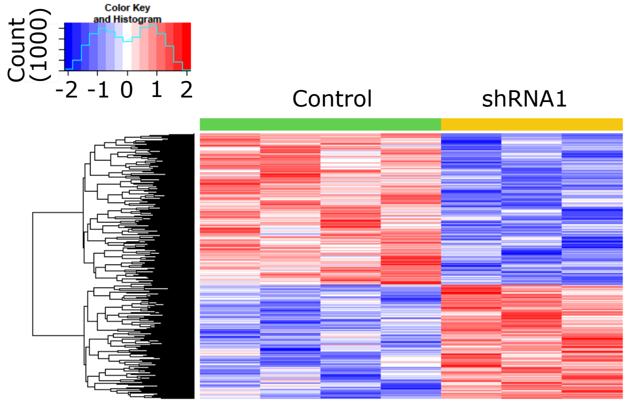

GSEA Hallmark Gene Sets

GSEA Hallmarks	# of Enriched Genes (total 178)	p-value
Estrogen Response Late	9	$3.12\ e^{-7}$
TNFA Signaling Via NFKB	8	$3.46\ e^{-6}$
UV Response DN	6	$4.74\ e^{-5}$
Inflammatory Response	6	$2.86\ e^{-4}$
UV Response Up	5	$7.28\ e^{-4}$
Epithelial Mesenchymal Transition	5	$2.06\ e^{-3}$
Estrogen Response Early	5	$2.06\ e^{-3}$
KRAS Signaling DN	5	$2.06\ e^{-3}$
IL6_JAK_STAT3 Signaling	3	$6.95\ e^{-3}$
Angiogenesis	2	$1.12\ e^{-2}$

EP300 shRNA2

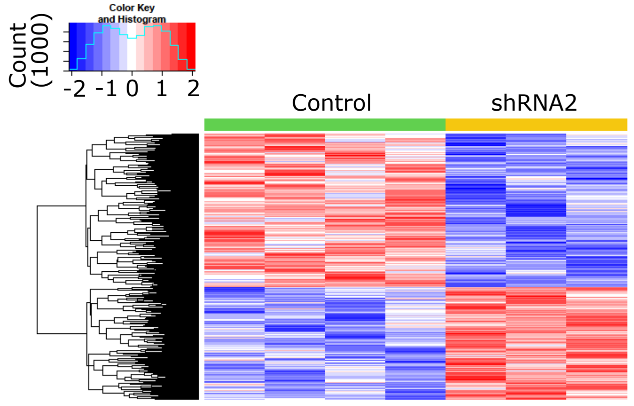

GSEA Hallmark Gene Sets

GSEA Hallmarks	# of Enriched Genes (total 144)	p-value
Estrogen Response Late	7	$8.57\ e^{-6}$
TNFA Signaling Via NFKB	6	$9.02\ e^{-5}$
UV Response DN	5	$1.8\ e^{-4}$
Epithelial Mesenchymal Transition	5	$8.09\ e^{-4}$
Allograft Rejection	4	$6.01\ e^{-3}$
Estrogen Response Early	4	$6.01\ e^{-3}$
KRAS Signaling DN	4	$6.01\ e^{-3}$
Angiogenesis	2	$7.46\ e^{-3}$
Hedgehog Signaling	2	$7.46\ e^{-3}$

Figure S4. A-485 and EP300 genetic knockdown commonly repress ER target genes in MCF-7 cells

(A) 24 h treatment with A-485 (3 μM) downregulates expression of ESR1 (n = 3), FASN (n = 3) and TFF1 (n = 2), as measured by RT-qPCR, in MCF-7 cells.

(B) EP300 genetic knockdown via two different shRNAs reduces ER target gene expression in MCF-7 cells (data from GEO GSE76200). A heatmap of the differentially expressed genes (p value < 0.05, |FC| ≥ 1.25) for each EP300 shRNA is shown on the left. The downregulated genes were analyzed for their involvement in biological pathways using the GSEA Hallmarks Gene Sets. The top statistically enriched pathways are shown in a table (right).

A

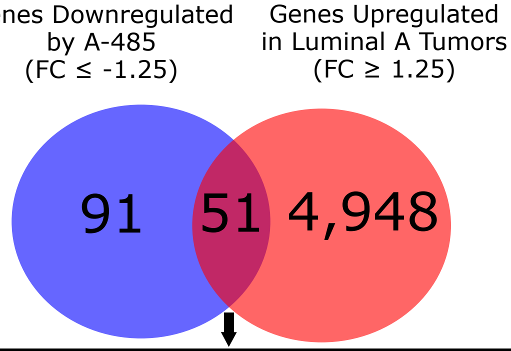

GSEA Hallmarks	# of Enriched Genes (total 51)	p-value
Estrogen Response Early	15	4.34e-23
Estrogen Response Late	14	3.8e-21

Estrogen Response Early

Gene Name	A-485 vs. DMSO	Luminal A Tumors vs. Normal
SLC7A5	↓	↑
DHCR7	↓	↑
XBP1	↓	↑
SLC9A3R1	↓	↑
CCND1	↓	↑
KRT19	↓	↑
CA12	↓	↑
FAM102A	↓	↑
TFF1	↓	↑
ANXA9	↓	↑
KRT8	↓	↑
KRT18	↓	↑
SLC7A2	↓	↑
SLC39A6	↓	↑
GFRA1	↓	↑

↑ Upregulated Expression

↓ Downregulated Expression

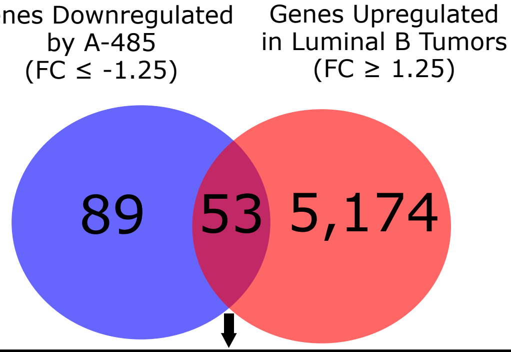

GSEA Hallmarks	# of Enriched Genes (total 53)	p-value
Estrogen Response Early	15	8.43e-23
Estrogen Response Late	14	7e-21

Estrogen Response Early

Gene Name	A-485 vs. DMSO	Luminal B Tumors vs. Normal
SLC7A5	↓	↑
DHCR7	↓	↑
XBP1	↓	↑
SLC9A3R1	↓	↑
CCND1	↓	↑
KRT19	↓	↑
CA12	↓	↑
FAM102A	↓	↑
TFF1	↓	↑
ANXA9	↓	↑
KRT8	↓	↑
KRT18	↓	↑
SLC7A2	↓	↑
SLC39A6	↓	↑
GFRA1	↓	↑

↑ Upregulated Expression

↓ Downregulated Expression

B

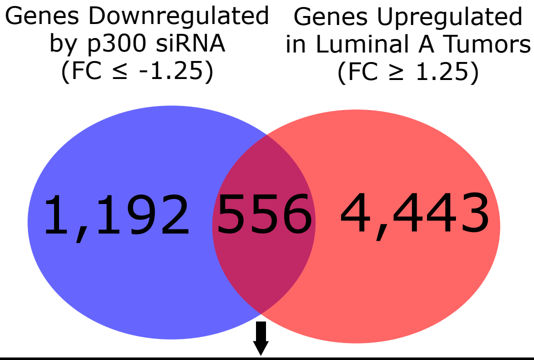

GSEA Hallmarks	# of Enriched Genes (total 555)	p-value
Estrogen Response Early	52	8.96e-51
Estrogen Response Late	46	2.5e-42

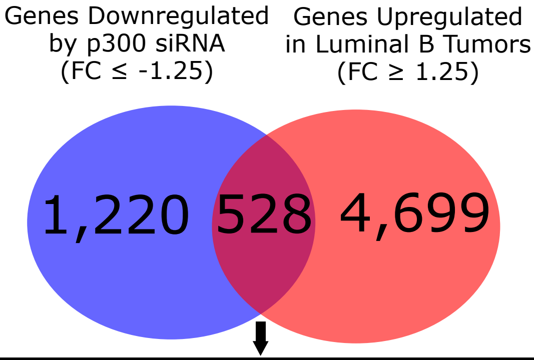

GSEA Hallmarks	# of Enriched Genes (total 527)	p-value
Estrogen Response Early	46	2.32e-43
Estrogen Response Late	41	1.48e-36

Figure S5: A-485 and EP300 KD reduce expression of genes that are upregulated in luminal BC

A) Genes downregulated by A-485 in MCF-7 cells are compared to genes that are upregulated in Luminal A tumors (left) and Luminal B tumors (right). The number of overlapping genes and the top two enriched pathways are shown. Tumor data is from the TCGA.

(B) Genes downregulated by p300 siRNA in MCF-7 cells are compared to genes that are upregulated in Luminal A tumors (left) and Luminal B tumors (right). The number of overlapping genes and the top two enriched pathways are shown. Tumor data is from the TCGA.

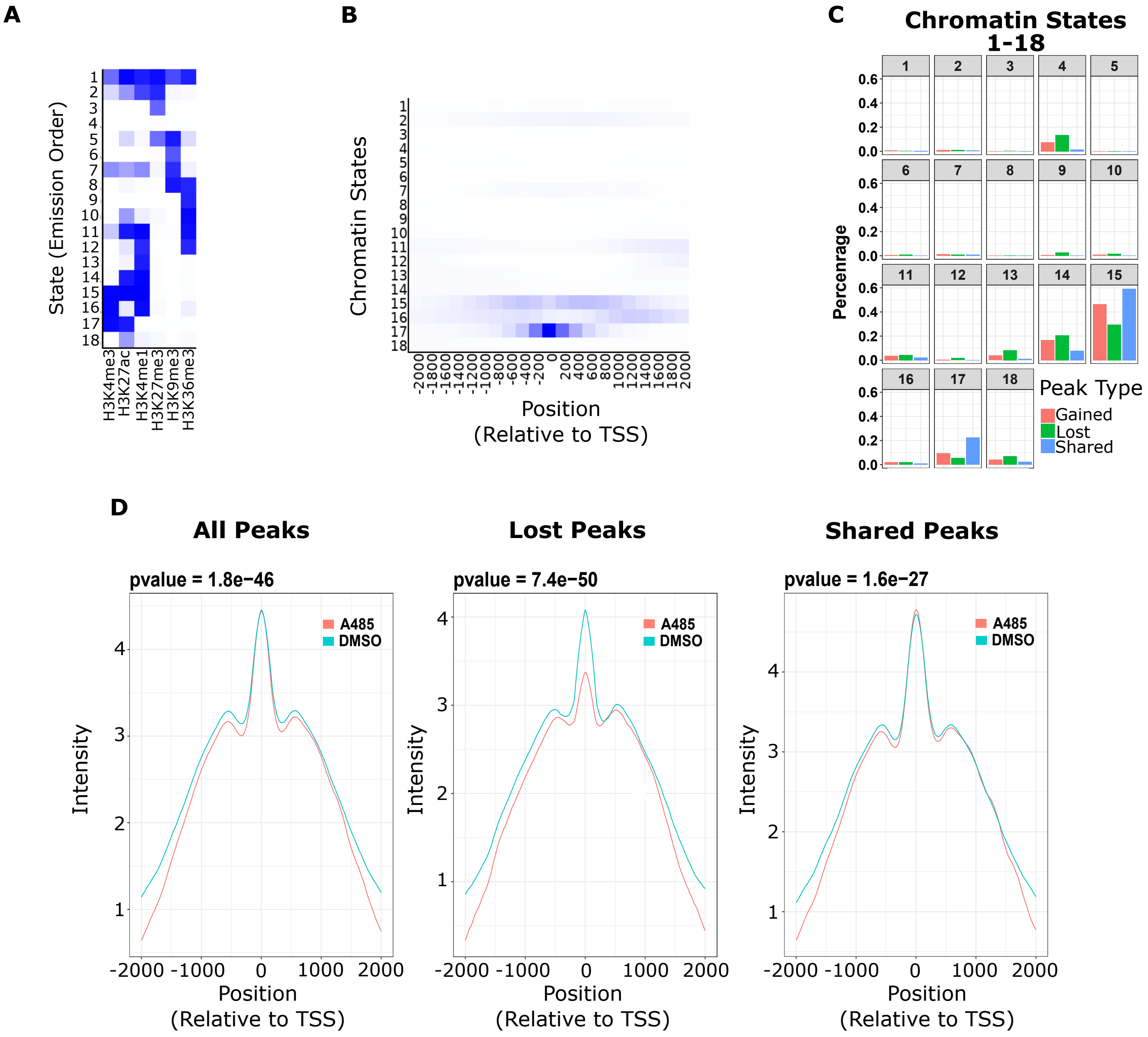

Figure S6. ChromHMM Analysis in MCF-7 cells

A) Six histone marks (namely H3K27ac, H3K27me3, H3K36me3, H3K4me1, H3K4me3, H3K9ac, H3K9me3 in MCF-7 cells, data from GEOGSE85158) were used to identify chromatin regions enriched with a different combination of these marks. ChromHMM model was trained using these chromatin marks and 18 states were identified. State 14 is defined as an Active Enhancer, State 15 as Flanking TSS and State 17 as an Active TSS.

(B) Enrichment of different chromatin states identified in (A) relative to the TSS.

(C) The distribution of H3K27ac peaks for the 18 chromatin states are shown for each of the peak types (Lost, Gained and Shared). Active regulatory regions of the genome (State 14, 15 and 17) are the most enriched states in our dataset.

(D) H3K27ac peak intensity at TSS regions defined by HomeR for each peak type.

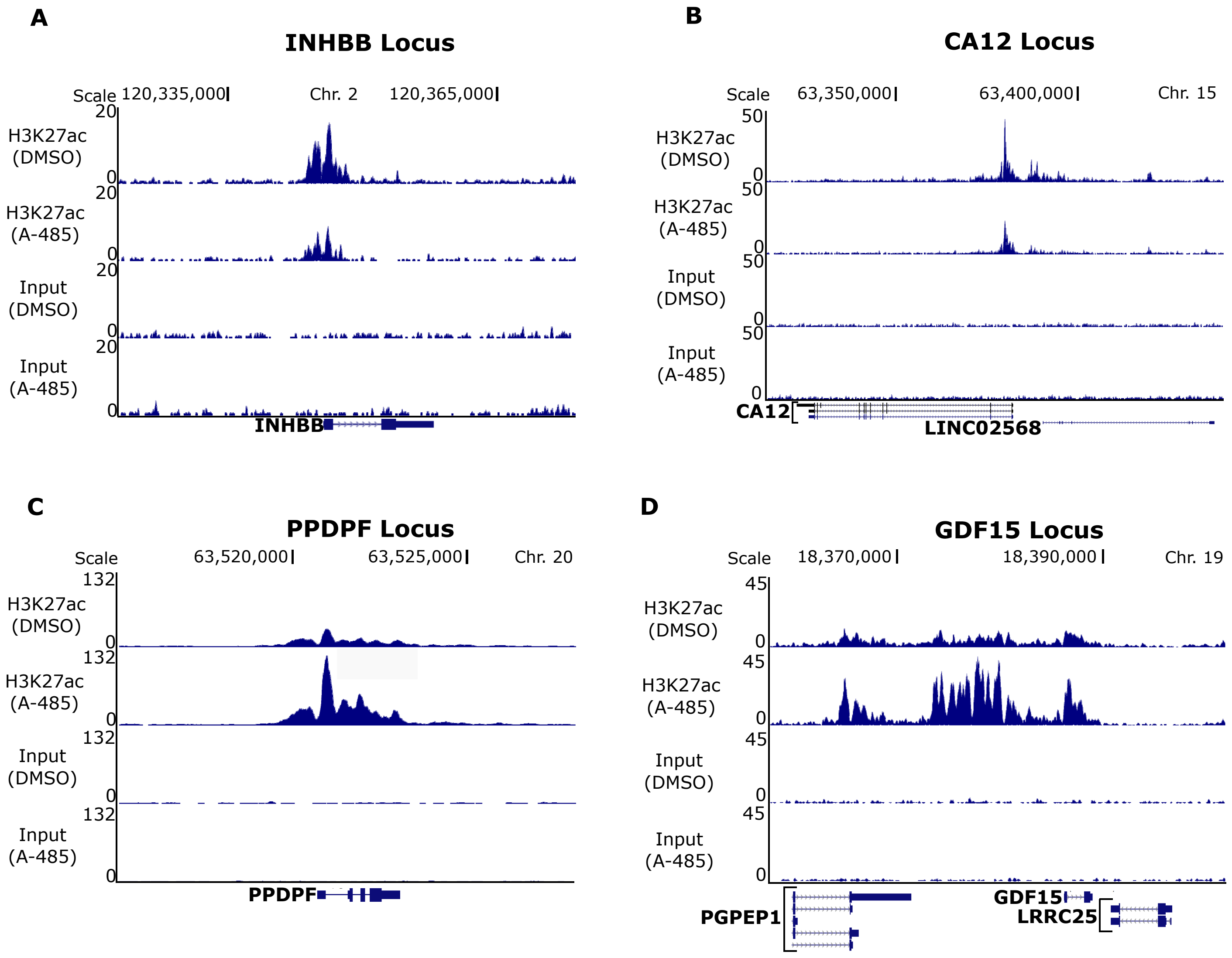

Figure S7. A-485 reduces H3K27ac at ER target genes but increases H3K27ac at a subset of genes

(A and B) UCSC genome browser tracks for the INHBB (A) and CA12 (B) locus. A-485 reduces H3K27ac intensity at the INHBB (A) and CA12 (B) promoter.

(C and D) UCSC genome browser tracks for the PPDPF (C) and GDF15 (D) locus. A-485 increases H3K27ac intensity at the PPDPF (C) and GDF15 (D) promoter.

A

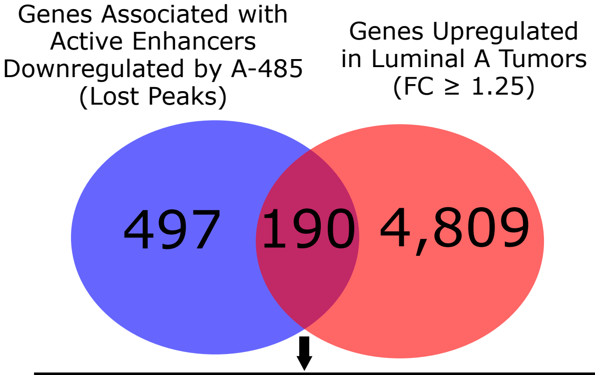

GSEA Hallmarks	# of Enriched Genes (total 190)	p-value
Estrogen Response Early	14	9.04e-13
Estrogen Response Late	9	5.15e-7

Estrogen Response Early

Gene Name	A-485 vs. DMSO	Luminal A Tumors vs. Normal
SEMA3B	↓	↑
SULT2B1	↓	↑
FLNB	↓	↑
RAB31	↓	↑
DHRS2	↓	↑
ANXA9	↓	↑
IGF1R	↓	↑
ABAT	↓	↑
RARA	↓	↑
KDM4B	↓	↑
WWC1	↓	↑
MLPH	↓	↑
THSD4	↓	↑
GREB1	↓	↑

↑ Upregulated Expression

↓ Downregulated Enhancer H3K27ac

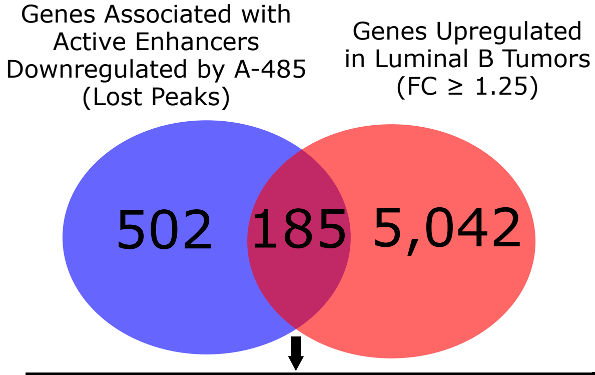

GSEA Hallmarks	# of Enriched Genes (total 185)	p-value
Estrogen Response Early	13	1.1e-11
Estrogen Response Late	9	4.11e-7

Estrogen Response Early

Gene Name	A-485 vs. DMSO	Luminal B Tumors vs. Normal
SULT2B1	↓	↑
FLNB	↓	↑
RAB31	↓	↑
DHRS2	↓	↑
ANXA9	↓	↑
IGF1R	↓	↑
ABAT	↓	↑
RARA	↓	↑
KDM4B	↓	↑
WWC1	↓	↑
MLPH	↓	↑
THSD4	↓	↑
GREB1	↓	↑

↑ Upregulated Expression

↓ Downregulated Enhancer H3K27ac

Figure S8: A-485 reduces H3K27ac at active enhancers associated with genes that are upregulated in luminal BC

(A) Genes with active enhancers that have reduced H3K27ac intensity in MCF-7 cells treated with A-485 (lost peaks category) are compared to genes that are upregulated in Luminal A tumors (left) and Luminal B tumors (right). The number of overlapping genes and their pathway enrichment are shown. Tumor data is from the TCGA.
